# Nexilin promotes calcium-dependent endo-lysosomal fission required for retrograde transport

**DOI:** 10.1186/s12964-025-02628-8

**Published:** 2026-01-09

**Authors:** Marie Bergundhaugen, Marte Sneeggen, Cinzia Progida

**Affiliations:** Department of Biosciences, University of Oslo, Oslo, Norway

## Abstract

Late endosomes and lysosomes (LE/Lys) are dynamic organelles primarily involved in the degradation of macromolecules within cells. Beyond this role, they are crucial for nutrient sensing, calcium signalling, cell proliferation and migration. The cytoskeleton is essential for the proper trafficking and function of LE/Lys. However, while the role of microtubules is well-established, the function of the actin cytoskeleton at LE/Lys is less understood. In this work, we performed an siRNA screen to identify actin regulators that play a role at LE/Lys. The screen revealed that the F-actin-binding protein nexilin regulates LE/Lys size as its depletion leads to their enlargement. We show that this is a consequence of inhibited LE/Lys fission and affects the retrograde transport from late endosomes to Golgi. Moreover, our data demonstrate that nexilin interacts with the small GTPase Rab7b and the lysosomal calcium channel TRPML1, and that TRPML1 activation rescues the LE/Lys enlargement caused by nexilin depletion. Taken together, our results indicate that nexilin mediates the interaction between LE/Lys and the acto-myosin cytoskeleton required for the calcium-dependent fission of these organelles.

**Supplementary Information:**

The online version contains supplementary material available at 10.1186/s12964-025-02628-8.

## Introduction

Late endosomes and lysosomes (hereafter referred to as LE/Lys) are dynamic organelles mainly known for their role in the degradation of macromolecules within the cell [1]. However, they are also important for other cellular processes such as nutrient sensing, calcium signalling, cell proliferation and migration [2–6]. Dysregulation of LE/Lys is therefore at the forefront of many diseases, such as lysosomal storage diseases, neurodegenerative disorders or cancer [7–9].

The cell’s cytoskeleton has an important role in ensuring correct trafficking and function of LE/Lys[10–12]. Microtubules, together with their motor proteins, have a well-established function in the regulation of LE/Lys positioning in the cell, which in turn influences their functions [6]. On the other side, the role of the actin cytoskeleton at LE/Lys is less characterized. In Dictyostelium, the actin surrounding LE/Lys has been suggested to prevent LE/Lys docking and fusion [13]. However, in macrophages, the actin assembled on LE/Lys or phagosomes is proposed to facilitate their docking and fusion [14–16]. Furthermore, it has been shown that LE/Lys recruit the actin nucleation machinery thus contributing to the movement of these organelles in Xenopus eggs [17].

At early endosomes, it is widely recognized that actin cytoskeleton mediates membrane fission. There, the Arp2/3 complex nucleates branched actin networks to promote cargo sorting and recycling [18, 19]. It has also been suggested that actin on early endosomes has a role in the formation of transport intermediates for early-to-late endosome transport required for endosomal maturation [20].

Endosomal fission is important for correct transport and recycling of endosomal cargo, maintenance of membrane lipid composition, and endosomal function [21, 22]. Endosomal fission is mediated by several components [23]: the endoplasmic reticulum marks the fission sites on the membrane [24], the microtubules together with dyneins or kinesins cause tubulation of the fission tubules [25], while the actin cytoskeleton acts together with myosins to generate membrane tension that is required for scission [18, 21, 26, 27]. Most of these studies demonstrate the mechanisms regulating membrane fission at early endosomes. The actin at LE/Lys is also proposed to have a role in membrane fission [28, 29], although the underlying mechanisms are less characterized.

To shed light on the role of the actin cytoskeleton at LE/Lys, we performed an siRNA screen targeting 48 different actin regulators, and assessed their effect on LE/Lys by microscopy. From the screen, we selected the F-actin-binding protein nexilin [30] as its knock down induced LE/Lys enlargement. Nexilin has previously been described to affect adhesion and migratory ability of HeLa cells [31]. More recently, nexilin has been shown to be important for the function of muscle cells, where it links calcium channels on the sarcoplasmic reticulum to RyR channels, affecting contractibility of cardiac cells [32]. Here, we find that nexilin, through the interaction with the lysosomal calcium channel TRPML1 (also known as MCOLN1), regulates endolysosomal calcium release and thus LE/Lys fission. Furthermore, we reveal that it also interacts with the LE/Lys small GTPase Rab7b and that its depletion, by preventing endosomal fission, affects Rab7b-mediated transport and possibly cargo recycling.

Together, our results shed light on a novel function of nexilin in regulating calcium-dependent LE/Lys dynamics, revealing a new mechanism of LE/Lys fission.

## Results

### An SiRNA screen identified nexilin as a regulator of late endosome/lysosome size

**Fig. 1:**
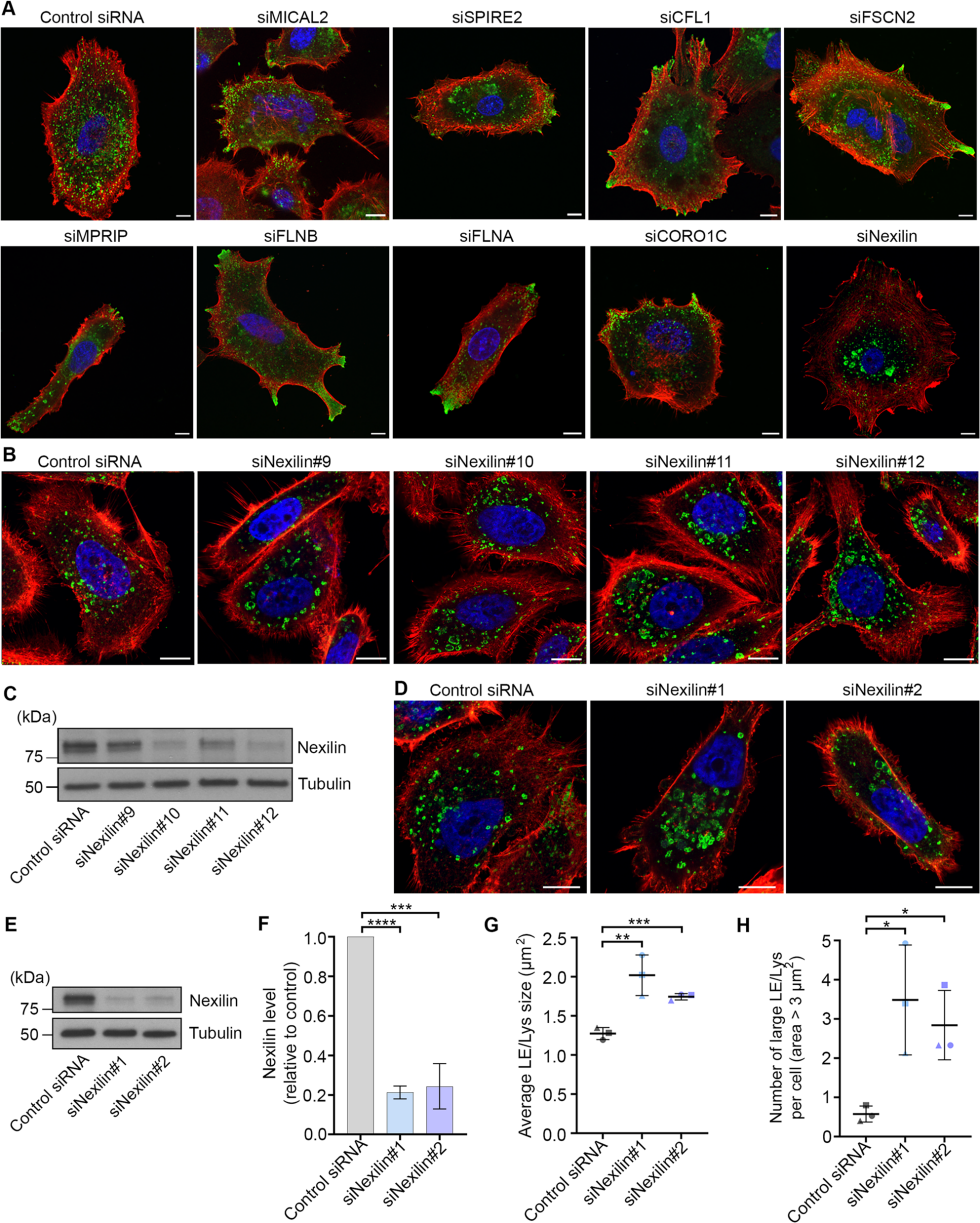
An siRNA screen identified Nexilin as a regulator of LE/Lys size. **A** PC3 cells were transfected with 48 pools of four different siRNAs per target gene or non-targeting control siRNAs for 72 h. Cells were then fixed and stained with rhodamine phalloidin (red), DAPI (blue), and an antibody against LAMP1 (green). Representative confocal images are shown for the nine actin regulators whose depletion consistently affected LE/Lys distribution in three independent screens in comparison to the control. Scale bar: 10 μm. **B** PC3 cells were transfected with four different siRNAs targeting nexilin or non-targeting control siRNA for 72 h. The cells were fixed and stained with rhodamine phalloidin (red), DAPI (blue), and an antibody against LAMP1 (green). Representative super-resolution confocal images are shown. Scale bar: 10 μm. **C** PC3 cells transfected with the indicated siRNAs targeting nexilin or non-targeting control siRNA were lysed and subjected to Western blot analysis using antibodies against nexilin and tubulin. **D** PC3 cells were transfected with two siRNAs targeting nexilin or non-targeting control siRNA for 72 h. The cells were fixed and stained with rhodamine phalloidin (red), DAPI (blue), and an antibody against LAMP1 (green). Scale bar: 10 μm. **E** PC3 cells transfected with the indicated siRNAs targeting nexilin or non-targeting control siRNA were lysed and subjected to Western blot analysis using antibodies against nexilin and tubulin. **F** Nexilin protein levels in PC3 cells transfected with control siRNA or siRNAs against nexilin were quantified using densitometry, normalized against the levels of tubulin, and plotted relative to the intensities in cells transfected with control siRNA. Data represents the mean ± s.d. from three independent experiments. **G** Quantification of average LE/Lys area (µm^2^) per cell in PC3 cells transfected with control siRNA or siRNAs against nexilin. Data represents the mean ± s.d. from three independent experiments (*n* = 45 cells in total per condition). **H** Quantification of the number of large LE/Lys with an area over 3 µm^2^ per cell in cells transfected with control siRNA or siRNAs against nexilin. Data represents the mean ± s.d. from three independent experiments (*n* = 45 cells in total per condition). * *p* < 0.05, ** *p* < 0.01 *** *p* < 0.001, **** *p* < 0.0001 (two-tailed unpaired Student’s *t*-test)

The actin cytoskeleton is present at several intracellular organelles, including the endoplasmic reticulum, mitochondria, early endosomes and LE/Lys [11, 20, 33, 34]. However, the role of actin at these organelles is not fully established. To shed light onto the role of the actin cytoskeleton on LE/Lys, we performed a RNAi screen using an siRNA library targeting 48 actin regulators. PC3 cells were transfected with pools containing four different siRNAs per target gene or non-targeting control siRNAs. 72 h after transfection, the cells were fixed and actin and LE/Lys were stained using rhodamine phalloidin and an antibody against LAMP1, respectively. Super-resolution confocal microscopy revealed that from the 48 different siRNAs, 9 showed a consistent different LE/Lys phenotype compared to the cells transfected with the control siRNAs, where 8 siRNA pools (MICAL2, SPIRE2, CFL1, FSCN2, MPRIP, FLNB, FLNA, CORO1C) showed LE/Lys repositioning towards the cell protrusions and one (NEXN, targeting nexilin) showed enlarged LE/Lys in the perinuclear region (Fig. 1A). To confirm these results and exclude off-target effects, we separately tested each of the four oligos present in the pools of the 9 targets that showed an altered LE/Lys phenotype. From the deconvolution screen, four of the targets showed inconsistent results (MICAL2, CFL1, FLNB, CORO1C), while a peripheral re-distribution of LE/Lys was confirmed upon knock down of SPIRE2, FSCN2, MPRIP, FLNA, and perinuclear and enlarged LE/Lys for nexilin (Fig. 1B). Nexilin was selected for further investigation as the only target from the screen that consistently resulted in the enlargement and clustering of LE/Lys. However, due to silencing efficiency variability (Fig. 1C, Supplementary Fig. 1 A) for several of the siRNAs from the screen, and potential off-target effects for the siRNA#12 due to sequence similarity with other genes, we further tested two additional siRNAs (siNexilin#1 and #2), targeting regions in the proximity of those targeted by siNexilin#10 and #12. siNexilin#1 and #2 caused the same enlargement and perinuclear localisation of LE/Lys as the siRNAs from the screen (Fig. 1D), with a silencing efficiency of about 80% (Fig. 1E-F, Supplementary Fig. 1 A), and were therefore used for follow-up studies.

Quantification of LE/Lys size revealed a significant, over 5-fold increase in the number of larger endosomes with an area over 3 µm^2^ upon knock down of nexilin, per cell (Fig. 1G-H). Incubation of cells depleted for nexilin with Magic Red, a probe for detecting altered Cathepsin B activity and frequently used to assess lysosomal function [35], reveal that nexilin depletion did not affect the functionality of these organelles (Supplementary Fig. 1B-D). Further, this enlargement was not observed for early endosomes (Supplementary Fig. 1E-F), indicating that nexilin has a specific role in the regulation of LE/Lys size.

### Nexilin interacts with the small GTPase Rab7b and regulates the size of Rab7b-positive LE/Lys

**Fig. 2:**
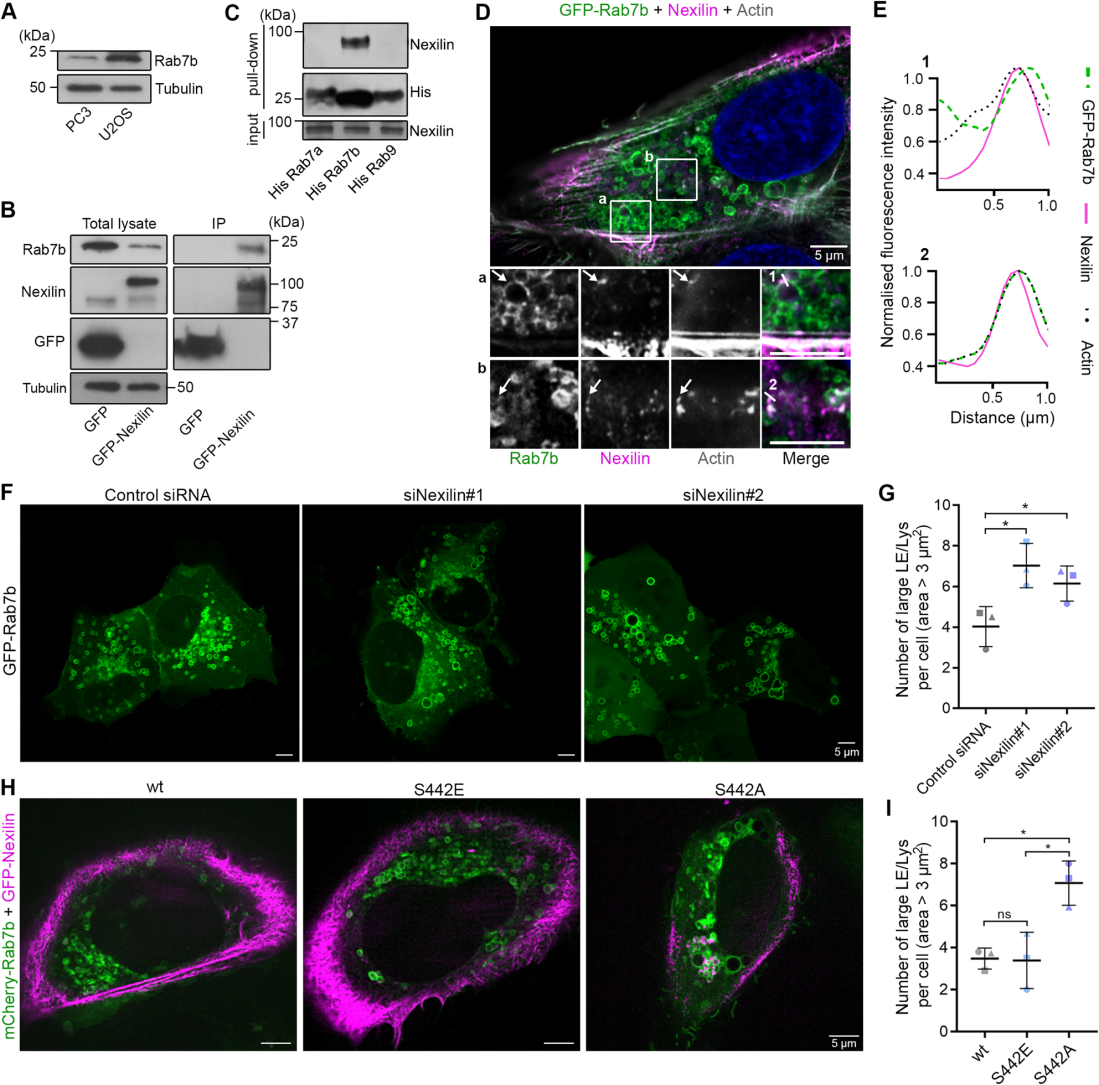
Nexilin interacts with Rab7b and regulates the size of Rab7b-positive LE/Lys. **A** Cell lysates from PC3 and U2OS cells were analysed through Western blot using antibodies against Rab7b and tubulin as loading control. **B** U2OS cells transiently transfected with GFP or GFP-nexilin were lysed and subjected to immunoprecipitation with GFP magnetic agarose beads. The total lysates and immunoprecipitated proteins were analysed by Western blot using the indicated antibodies. **C** Bacterially expressed and purified His-Rab7a, His-Rab7b, and His-Rab9 were incubated with HeLa cell lysates. Proteins were pulled down using cobalt-coated magnetic beads and subjected to Western blot analysis using the indicated antibodies. **D** U2OS cells transiently transfected with GFP-Rab7b (green) were fixed and stained with rhodamine phalloidin (grey) and an antibody against nexilin (magenta) and imaged using a super-resolution confocal microscope. White square indicates the magnified areas, arrows point to areas of colocalisation. Scale bar: 5µm. **E** Fluorescence intensity profile of Rab7b (green), nexilin (magenta) and actin (black), measured along the indicated lines and normalised to the max fluorescence value in their respective channels. **F** U2OS cells were treated with control siRNA or different siRNAs against nexilin and transiently transfected with GFP-Rab7b before live cell imaging. Scale bar: 5 µm. **G** Quantification of the number of Rab7b-positive LE/Lys with an area larger than 3 µm^2^ per cell. Data represents the mean ± s.d. from three independent experiments (n = 60 cells in total per condition), two-tailed, unpaired Student’s *t*-test was applied for statistical analysis. **H** U2OS cells were co-transfected with mCherry-Rab7b (green), and either GFP-nexilin wild type (wt), GFP-nexilin S442A (phosphorylation-mimic mutant), or GFP-nexilin S442A (phosphorylation-null mutant), shown in magenta. Scale bar: 5 µm. **I** Quantification of number of Rab7b-positive LE/Lys with area over 3 μm^2^ per cell in cells co-transfected with mCherry-Rab7b and either GFP-nexilin wild type (wt), GFP-nexilin S442A, or GFP-nexilin S442A. Data represents the mean ± s.d. from three independent experiments (n = 30 cells in total per condition), one-way ANOVA, followed by Tukey’s post-hoc test was applied for statistical analysis. ns non-significant, * p < 0.05

In a previously performed yeast two-hybrid screen using the LE/Lys small GTPase Rab7b as bait [36], we identified nexilin between the putative interaction partners. To confirm the interaction, we performed co-immunoprecipitation experiments in U2OS cells transiently transfected with GFP or GFP-nexilin, as these cells express higher levels of Rab7b than PC3 (Fig. 2A). GFP-nexilin, and not the GFP control, co-immunoprecipitated with endogenous Rab7b, indicating that nexilin indeed interacts with Rab7b (Fig. 2B). The specificity of the interaction was also assessed through pulldown experiments, using LE/Lys His-tagged Rab proteins expressed and purified from bacteria to evaluate the ability of different Rabs to pull down nexilin from cell extracts. Only Rab7b was able to pull down nexilin, indicating that the interaction is specific for this small GTPase (Fig. 2C). Next, we investigated whether nexilin localizes to Rab7b-positive LE/Lys by confocal super-resolution microscopy. U2OS cells were transiently transfected with GFP-Rab7b, fixed and stained for nexilin and actin using an antibody and rhodamine phalloidin, respectively. Nexilin puncta were indeed present on actin clusters on Rab7b-positive endosomal membranes (Fig. 2D-E).

Next, we sought to determine if nexilin knock down altered Rab7b-endosomal size similarly to what we observed for LAMP1-positive LE/Lys, as Rab7b is present on these compartments [37, 38]. U2OS cells were knocked down for nexilin, and transfected with GFP-Rab7b before live cell imaging using a confocal spinning disk microscope (Fig. 2F). In line with our previous observations, LE/Lys become enlarged upon nexilin knock down compared to control siRNA-treated cells, with an increased number of Rab7b-positive endosomes larger than 3 µm^2^ in area (Fig. 2G). The same effect was further observed in both PC3 and HeLa cells, indicating that the effect of nexilin depletion on LE/Lys size is independent of the cell type (Supplementary Fig. 2A-E).

A previous study reported phosphorylation of nexilin on Ser 437 by CLK4 in mice [39]. The phosphorylation activates nexilin, while leaving Ser 437 unphosphorylated renders nexilin inactive. To assess whether inactivation of nexilin by inhibiting phosphorylation phenocopies nexilin knock down, we generated a nexilin phosphomimic mutant by replacing the corresponding Ser in the human nexilin with Glu (S442A), and a phosphorylation-null mutant by replacing Ser 442 with Ala (S442A, Supplementary Fig. 2 F), as this phosphorylation site was previously described as evolutionarily conserved across species [39].

U2OS cells were co-transfected with either GFP-tagged nexilin wild type, the S442A mutant, or S442A mutant, together with mCherry-Rab7b, and imaged live using a spinning disk confocal microscope (Fig. 2H). The colocalization of Rab7b with the different nexilin mutants did not change significantly (Supplementary Fig. 2G). However, both the wild type and the phosphorylation-mimic mutant S442A showed a similar phenotype, while the phosphorylation-null mutant of nexilin resulted in increased number of enlarged endosomes (Fig. 2I). These results indicate that the phosphorylation-null mutant indeed mirrors nexilin knock down, additionally confirming that nexilin inactivation triggers enlargement of Rab7b-positive endosomes. Taken together, these findings indicate that nexilin interacts with Rab7b and confirms its role in regulating LE/Lys size.

### Nexilin affects myosin II activation and regulates LE/Lys size by interacting with the lysosomal calcium channel TRPML1

**Fig. 3:**
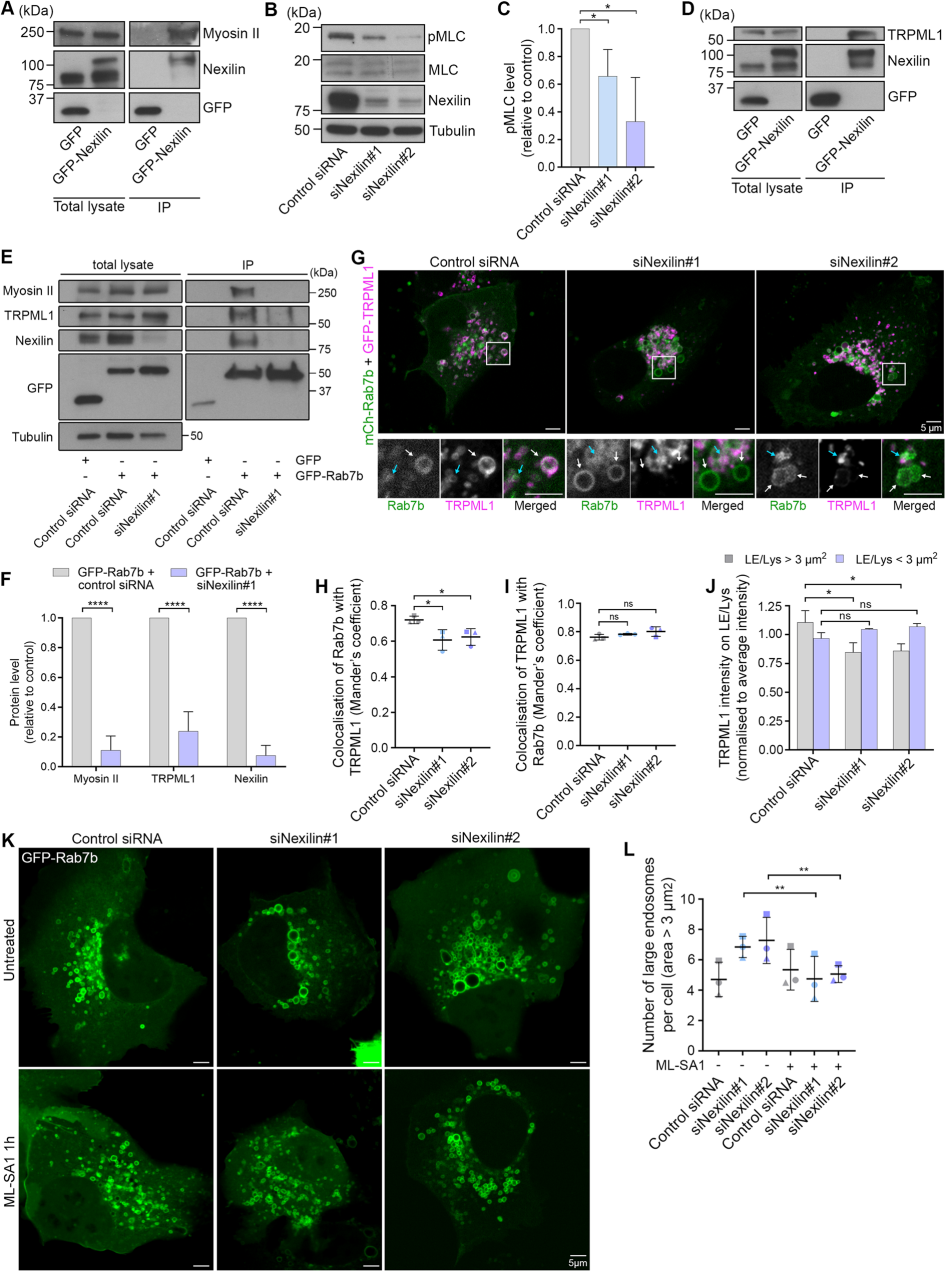
Nexilin regulates LE/Lys size through the interaction with the lysosomal Ca^2+^ channel TRPML1. **A** U2OS cells transiently transfected with either GFP or GFP-Nexilin were subjected to immunoprecipitation with GFP magnetic agarose beads. The total lysates and immunoprecipitated proteins were analysed through Western blot using the indicated antibodies. **B** U2OS cells depleted for nexilin by siRNA transfection were lysed and analysed by Western blot using the indicated antibodies. **C** Quantification of the levels of phosphorylated myosin light chain on Ser 19 (pMLC). The intensity of the bands from the Western blots were normalised to the total myosin light chain (MLC) levels, using tubulin as loading control. Data represents the mean ± s.d. from three independent experiments. Two-tailed, unpaired Student’s *t*-test was applied for statistical analysis. **D** U2OS cells were transiently transfected with either GFP or GFP-nexilin and subjected to immunoprecipitation with GFP magnetic agarose beads. The total lysates and immunoprecipitated proteins were analysed through Western blot using the indicated antibodies. **E** U2OS cells treated with either control siRNA or siRNA targeting nexilin were transiently transfected with either GFP or GFP-Rab7b. The lysates were subjected to immunoprecipitation with GFP magnetic agarose beads and analysed through Western blot, using the indicated antibodies. **F** Quantification of the levels of myosin II, TRPML1, or nexilin in the indicated samples immunoprecipitated using GFP magnetic agarose beads. Intensity of the bands from Western blots were normalised to the immunoprecipitated GFP-Rab7b levels, and plotted relative to the control condition. Data represents the mean ± s.d. from three independent experiments. Two-tailed, unpaired Student’s *t*-test was applied for statistical analysis. **G** U2OS cells were treated with control siRNA or siRNAs targeting nexilin and transiently co-transfected with mCherry-Rab7b and GFP-TRPML1 before live cell imaging. Blue and white arrows points to smaller and larger endosomes (under or over 3 µm^2^ in area), respectively. Scale bar: 5 μm. The graphs represent colocalisation analysis of: **H** Rab7b with TRPML1, **I** TRPML1 with Rab7b, analysed by calculating Mander’s coefficient in ImageJ. Data represents the mean ± s.d. for three independent experiments (*n* = 45 cells in total per condition). Two-tailed, unpaired student’s *t*-test was applied for statistical analysis. **J** The graph displays distribution of TRPML1 mean intensity on large (area > 3 µm^2^) and small (area < 3 µm^2^) LE/Lys, normalised to the total mean intensity of TRPML1 per cell, upon nexilin knock down. Two-tailed, unpaired Student’s *t*-test was applied for statistical analysis. **K** U2OS cells were treated with control siRNA or siRNAs targeting nexilin and transiently transfected with GFP-Rab7b before live cell imaging. The same cells were then treated with 20 µM of the TRPML1 agonist ML-SA1 for 1 h before live cell imaging. Scale bar: 5 μm. **L** Quantification of the number of Rab7b-positive endosomes larger than 3 µm^2^ in control cells and cells knocked down for nexilin, with and without ML-SA1 treatment. Data represents mean ± s.d. from three independent experiments (*n* ≥ 58 cells in total per condition). Two-way ANOVA, followed by Šidák post-hoc test was applied for statistical analysis. ns non-significant, * *p* < 0.05, ** *p* < 0.01, **** *p* < 0.0001

Similar enlargement of Rab7b-positive endosomes has previously been reported upon inhibition of myosin II, as consequence of the direct interaction between Rab7b and myosin II^36^. We therefore sought to determine if nexilin is part of this complex and interacts with myosin II. Nexilin was immunoprecipitated with GFP-trap magnetic agarose beads in U2OS cells transfected with GFP-nexilin, and myosin II was co-immunoprecipitated with it, revealing that nexilin indeed interacts with myosin II (Fig. 3A).

To further investigate the link between Rab7b, nexilin and myosin II, we examined whether nexilin knock down, similarly to Rab7b depletion [36, 40], affects the phosphorylation of myosin II light chain, as phosphorylation of Ser 19 activates this motor protein [41]. Interestingly, we detected a significant reduction in the phosphorylation levels of myosin light chain upon nexilin knock down (Fig. 3B-C), suggesting that nexilin knock down inhibits myosin II activation. Similarly, expression of the nexilin phosphorylation-null mutant S442A significantly decreased the phosphorylation of myosin light chain, while the presence of the phosphorylation-mimic mutant S442A increased it (Supplementary Fig. 3A-B). This further confirms the role of nexilin in regulating the phosphorylation state and activation of myosin II.

Intriguingly, the lysosomal calcium channel TRPML1 is involved in the regulation of myosin II phosphorylation, and it interacts with both Rab7b and myosin II [3, 40]. Therefore, we next investigated if nexilin plays a role in this pathway. First, we evaluated if nexilin interacts with TRPML1 by performing co-immunoprecipitation experiments using GFP-trap beads on U2OS cells transfected with GFP-nexilin. GFP-nexilin was able to co-immunoprecipitate endogenous TRPML1, indicating an interaction between the two proteins (Fig. 3D). To then assess the relationship of nexilin with the complex consisting of Rab7b, myosin II and TRPML1, we performed immunoprecipitation experiments using GFP-trap magnetic agarose beads in U2OS cells transfected with control siRNA or siRNA targeting nexilin, and transiently transfected with GFP or GFP-Rab7b. The results revealed that the interactions of Rab7b with myosin II and TRPML1 were decreased upon nexilin knock down (Fig. 3E-F), indicating that nexilin is required for the proper assembly of the complex.

As nexilin knock down appeared to reduce the interaction between Rab7b and TRPML1, we sought to confirm this effect using microscopy. U2OS cells were knocked down for nexilin and transiently co-transfected with mCherry-Rab7b and GFP-TRPML1 (Fig. 3G). Interestingly, the colocalization of Rab7b with TRPML1 significantly decreased upon nexilin knock down (Fig. 3G-H). While TRPML1 still localised to Rab7b-positive endosomes (Fig. 3G, I), there were Rab7b-positive endosomes devoid of TRPML1 upon nexilin knock down (Fig. 3G), in line with our co-immunoprecipitation results (Fig. 3E-F). Intriguingly, less TRPML1 was present on the larger Rab7b-positive endosomes (larger than 3 µm^2^ in area) in cells knocked down for nexilin compared to control cells, while smaller endosomes remained TRPML1-positive in all conditions (Fig. 3J).

It has previously been suggested that the release of Ca^2+^ from TRPML1 regulates lysosome size [42]. Therefore, to determine if the lack of TRPML1 on the enlarged LE/Lys could explain the phenotype upon nexilin knock down, we used the TRPML1 agonist ML-SA1 [43, 44] to see if the activation of the Ca^2+^ channel restores the size of LE/Lys. U2OS cells knocked down for nexilin and subsequently transfected with GFP-tagged Rab7b were incubated with 20 µM ML-SA1 for 1 h, as previously described [42]. We first assessed lysosomal pH by staining the cells with LysoSensor to ensure that ML-SA1 did not cause lysosomal damage (Supplementary Fig. 3 C). Next, we measured lysosomal size. ML-SA1 treatment recovered the size of Rab7b-positive endosomes, indicating that nexilin silencing reduces the activity of TRPML1 thus resulting in LE/Lys enlargement (Fig. 3K-L).

Taken together, these results indicate that nexilin interacts with and plays a role in regulating myosin II and TRPML1 activity at LE/Lys.

### Nexilin regulates LE/Lys calcium dynamics

**Fig. 4:**
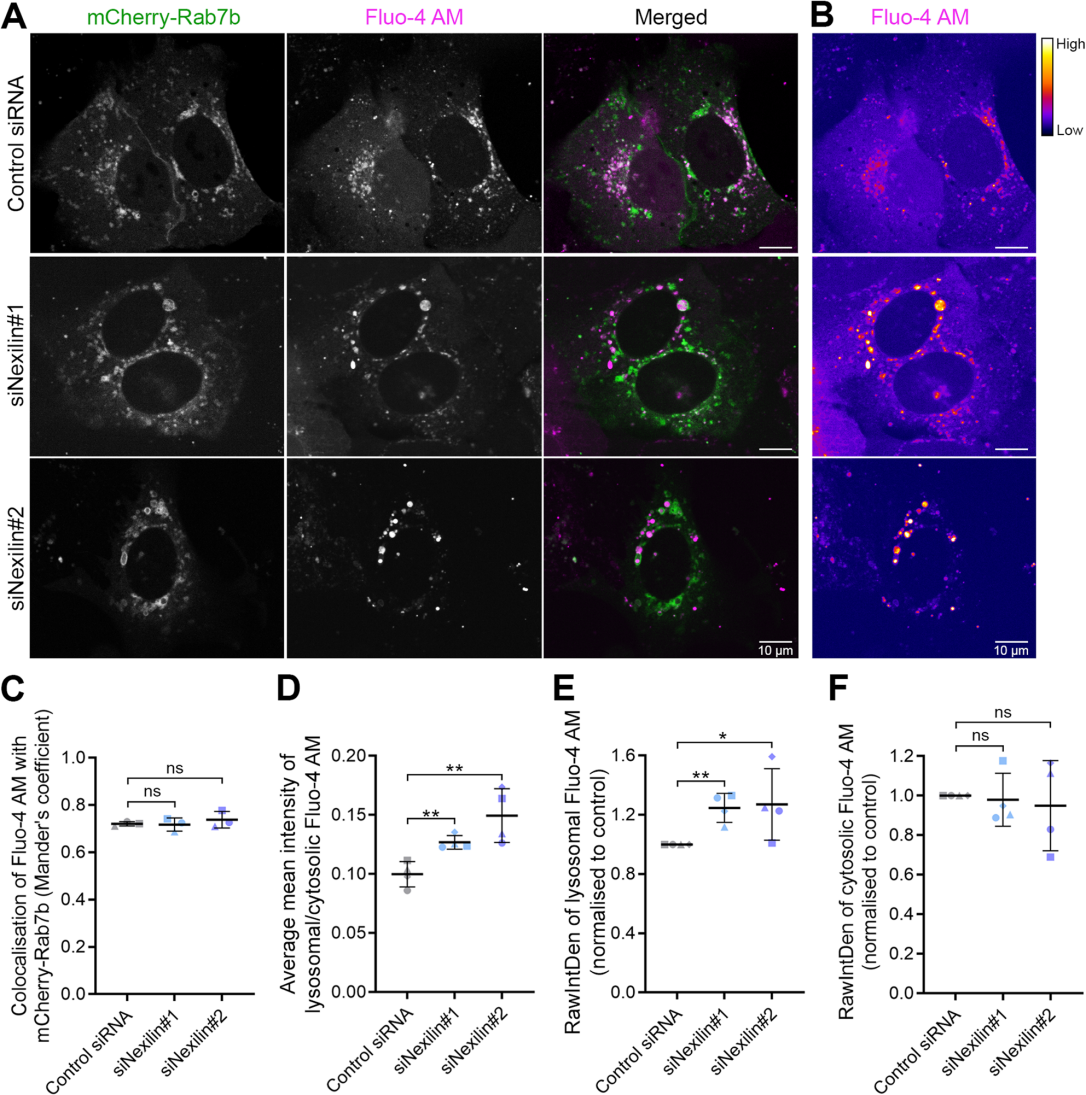
Nexilin knock down alters LE/Lys calcium dynamics. **A** U2OS cells treated with control siRNAs or siRNAs against nexilin and transfected with mCherry-Rab7b were incubated with Fluo-4 AM for 30 min before live cell imaging. Scale bar: 10 μm. **B** Intensity map showing Fluo-4 AM intensity for the cells shown in A). Scale bar: 10 μm. **C** Quantification of colocalisation of Fluo-4 AM with Rab7b using ImageJ to obtain Mander’s coefficient. Data represents mean ± s.d. from three independent experiments (*n* = 30 cells in total per condition). **D** Ratio of the average mean intensity of Fluo-4 AM fluorescence in LE/Lys over the cytosol. Data represents mean ± s.d. from four independent experiments (*n* ≥ 78 cells in total per condition). The graphs represent quantifications of raw integrated density of Fluo-4 AM in (**E**) LE/Lys or (**F**) cytosol in cells depleted for nexilin, normalised to the control condition. Data represents mean ± s.d. from four independent experiments (*n* ≥ 78 cells in total per condition). ns non-significant, * *p* < 0.05, ** *p* < 0.01 (two-tailed unpaired Student’s *t*-test)

As our results suggest that nexilin is involved in regulating TRPML1 activity, we next investigated if LE/Lys calcium dynamics are altered upon nexilin knock down using the fluorescent calcium-labelling probe Fluo-4 AM [45]. U2OS cells were treated with control siRNA or siRNAs targeting nexilin, transfected with mCherry-Rab7b, and incubated with Fluo-4 AM for 30 min before imaging. Fluo-4 AM displayed high colocalisation with Rab7b-positive LE/Lys, which was not affected by nexilin depletion (Fig. 4A, C). However, the intensity of Fluo-4AM in LE/Lys increased upon nexilin depletion compared to the cells treated with non-targeting control siRNA (Fig. 4B). Quantification of Fluo-4 AM intensity confirmed that the ratio between LE/Lys and cytosolic calcium significantly increased upon nexilin knock down (Fig. 4D), possibly as consequence of an increase in lysosomal calcium (Fig. 4E-F). Reintroduction of an siRNA-resistant BFP-nexilin construct in cells silenced for nexilin was able to decrease the LE/Lys calcium to levels similar to the control (Supplementary Fig. 4A-B). These results suggest that calcium accumulates in LE/Lys upon nexilin knock down.

Live cell imaging of cells stained with Fluo-4 AM revealed that in cells silenced for nexilin, addition of ML-SA1 could indeed stimulate the release of calcium from the LE/Lys, triggering tubulation and fission events, thus resulting in smaller LE/Lys (Supplementary Video 2).

### Nexilin knock down prevents the fission of Rab7b-positive endosomes, delaying Rab7b-mediated retrograde transport

Rab7b has previously been described to mediate retrograde transport from late endosomes to the Golgi [37, 38]. To determine if the LE/Lys defects caused by nexilin knock down affect Rab7b-mediated transport, we took advantage of fluorescently labelled Cholera toxin subunit B (CTxB), as CTxB retrograde transport is dependent on Rab7b [37]. U2OS cells treated with control siRNA, siRNA against nexilin or siRNA against nexilin and later transfected with siRNA-resistant BFP-nexilin, were incubated with Alexa Fluor 555 CTxB for 45 min and chased for 30 min. The cells were fixed and stained for the Golgi marker GM130 before imaging to determine CTxB retrograde trafficking to the Golgi.

**Fig. 5:**
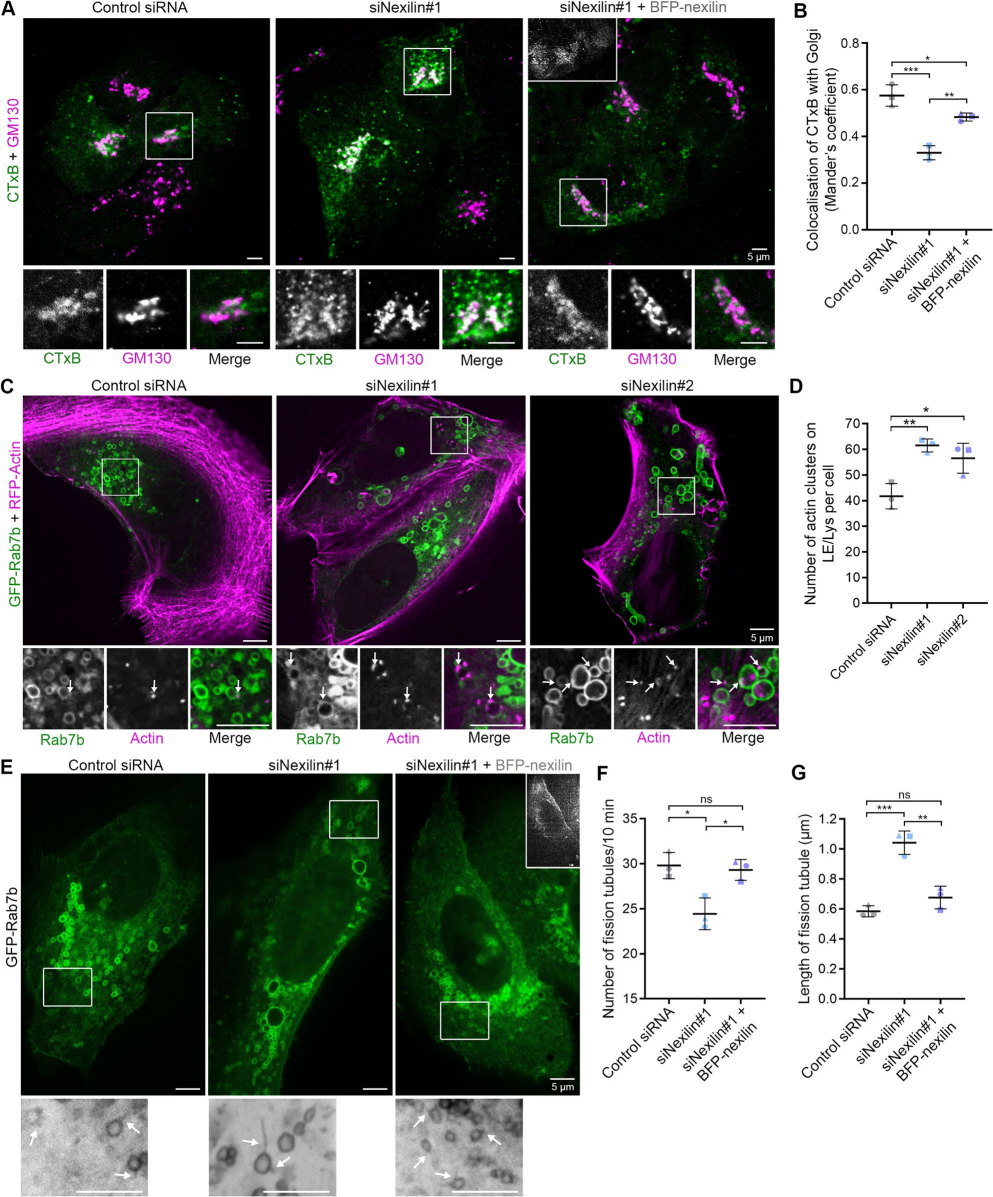
Nexilin depletion alters cholera toxin B retrograde transport to the Golgi and prevents LE/Lys fission. **A** U2OS cells were transfected with control siRNA, siRNA against nexilin, or siRNA against nexilin and then transfected with siRNA-resistant BFP-nexilin, and incubated with 4 µg/ml cholera toxin subunit B conjugated with AlexaFluor-555 (green) for 45 min, then chased for 30 min before fixation and staining using a primary antibody against GM130 (magenta). Inset image shows BFP-nexilin, enhanced by immunostaining with an antibody against nexilin, white squares indicate magnified areas. Scale bar: 5 $\:\mu\:$m. **B** Quantification of colocalization of CTxB with GM130 using ImageJ to obtain Mander’s coefficient. Data represents mean ± s.d. from three independent experiments (*n* ≥ 53 cells in total per condition). One-way ANOVA, followed by Tukey’s post-hoc test was applied for statistical analysis. **C** U2OS cells treated with control siRNA or siRNAs against nexilin and transiently co-transfected with LifeAct-RFP and GFP-Rab7b before live-cell imaging using a super-resolution spinning disk microscope. White squares indicate the magnified area. Arrows point to areas of colocalization. Scale bar: 5 μm. **D** Quantification of the number of actin clusters on Rab7b-positive endosomes per cell. Data represents mean ± s.d. from three independent experiments (*n* = 45 cells in total per condition). Two-tailed, unpaired Student’s *t*-test was applied for statistical analysis. **E** U2OS cells were treated with control siRNA, siRNAs against nexilin, or siRNA against nexilin and then transfected using siRNA-resistant BFP-nexilin, and transiently transfected with GFP-Rab7b before live cell imaging was performed every 10 s for 15 min. Inset image shows BFP-nexilin (grey), white squares indicate the magnified area. Arrows point to fission tubules. **F** The graph represents the average number of Rab7b-positive fission tubules per cell detected over 10 min (*n* = 24 cells in total per condition), one-way ANOVA followed by Tukey’s post-hoc test was applied for analysis. **G** Quantification of the average length of the fission tubules (µm) per cell (*n* = 24 cells in total per condition), one-way ANOVA followed by Tukey’s post-hoc test was applied for analysis. ns non-significant, * *p* < 0.05, ** *p* < 0.01, *** *p* < 0.001

About 60% of the internalized CTxB colocalised with the Golgi in cells transfected with control siRNA (Fig. 5A, B). Interestingly, in cells knocked down for nexilin, CTxB was present in vesicles spread through-out the cell, with a 45% reduction of colocalization of CTxB with GM130 compared to the control cells, which was largely rescued by reintroducing siRNA-resistant nexilin (Fig. 5A, B). A similar reduction in colocalization of CTxB with GM130 was also measured upon depletion of nexilin using a different siRNA (siNexilin #2), (Supplementary Fig. 5A-B). These results indicate that nexilin knock down indeed alters CTxB transport, suggesting that nexilin has a role in Rab7b-mediated endosomal cargo transport.

The enlargement of Rab7b-positive LE/Lys and altered CTxB transport upon nexilin depletion, made us wonder if nexilin plays a role in regulating fusion or fission of LE/Lys. The actin cytoskeleton is heavily involved in both of these processes [20, 21, 46], and because nexilin is an actin-interacting protein [30], we investigated if nexilin knock down affects actin localisation on LE/Lys. To test this, cells were depleted for nexilin and transiently co-transfected with LifeAct-RFP together with GFP-Rab7b (Fig. 5C). Interestingly, cells depleted for nexilin showed an increased presence of actin clusters on LE/Lys. Object-based colocalization analysis confirmed that the number of actin clusters present on Rab7b-positive LE/Lys increased upon nexilin depletion (Fig. 5D).

We next investigated if these actin clusters were positive for the Arp2/3 complex. The Arp2/3 complex promotes actin polymerisation [47, 48], including the formation of branched actin on actin clusters on the early endosomal membrane [20], and has been associated to regulation of endosomal fission events [18–21]. U2OS cells knocked down for nexilin were transiently transfected with GFP-Rab7b, fixed and stained for Arp3 and actin, using an antibody and rhodamine phalloidin, respectively. Arp3 was indeed detected on actin clusters on Rab7b-positive endosomes (Supplementary Fig. 5 C), and similarly to the actin clusters, the number of Arp3-positive clusters on Rab7b-positive endosomes increased upon nexilin depletion (Supplementary Fig. 5D).

The actin present on endosomes has been suggested to remodel endosomal membranes [20, 21, 47]. In addition, nexilin interacts with TRPML1 and myosin II, both implicated in regulating membrane fission [27, 42, 49]. Therefore, to verify if the lack of nexilin prevents LE/Lys fission, we performed live imaging of cells treated with control siRNA or siRNAs against nexilin immediately after transfection with GFP-Rab7b, imaging every 5 min for 15 h (Supplementary Fig. 5E, Supplementary Fig. 6, Supplementary Video 1). In cells depleted for nexilin, Rab7b-positive LE/Lys continued to fuse over time (Supplementary Fig. 6, Supplementary Video 1), with a significant reduction of fission events detected (Supplementary Fig. 5E-F, Supplementary Video 1). To better characterize the fission events and ensure to account for fast-occurring fission, we next employed rapid super-resolution imaging. Cells treated with control siRNA, siRNA against nexilin, or siRNA against nexilin and then transfected with an siRNA-resistant BFP-nexilin construct, were transiently transfected with GFP-Rab7b, and imaged every 10 s using a super-resolution spinning disk microscope (Fig. 5E). Quantification of the number of fission tubules confirmed a decrease upon nexilin depletion, which was rescued by reintroducing siRNA-resistant nexilin (Fig. 5F). Furthermore, the fission tubules were 1.8-fold longer in cells depleted for nexilin than in the control, and re-introduction of nexilin was able to rescue the length of the fission tubules (Fig. 5G), supporting a role for nexilin in mediating LE/Lys fission.

Taken together, our results indicate that nexilin knock down affects Rab7b-mediated trafficking by hampering LE/Lys fission.

## Discussion

The role of the acto-myosin cytoskeleton at LE/Lys is poorly understood. Here, we performed an siRNA screen targeting known actin regulators, and identified the actin-interacting protein nexilin as a modulator of LE/Lys size. Nexilin has previously been shown to regulate cell migration and adhesion [31, 50, 51], muscle contractility [52–54] and calcium homeostasis in cardiac muscle cells [32], but apart from that, its role in the cell is not well characterized. Our results reveal that nexilin is present at the LE/Lys, where it interacts with the small GTPase Rab7b, and its depletion increases LE/Lys size.

Interestingly, Rab7b has been shown to interact directly with the actin motor protein myosin II, bridging the actin cytoskeleton to the LE/Lys, and myosin II or Rab7b knock down both increase LE/Lys size [36, 40]. Our results indicate that nexilin is involved in regulating this interaction, as it co-immunoprecipitates both proteins and its depletion reduces the Rab7b-myosin II interaction. Nexilin knock down further affects myosin II activity by reducing the phosphorylation of its light chain, which is important for the activation of this motor protein [41]. Thus, nexilin depletion may prevent myosin II binding to Rab7b by hindering myosin II activation.

Nexilin is phosphorylated on Ser-442, which has been previously reported to regulate its activity [39]. Here, we observed that transfection with the phospho-ablative mutant S442A mimicked nexilin depletion by inducing LE/Lys enlargement and reduction of myosin II phosphorylation, in contrast to the phospho-mimic mutant S442A, which did not alter LE/Lys size, but increased the levels of phosphorylated myosin II light chain, confirming the role of nexilin in regulating myosin II activation. These results are in line with previous speculations suggesting that nexilin performs its function when phosphorylated [39], and indicate that the phosphorylation site in position 442 of nexilin is important for its activity. They further imply that a functional consequence of nexilin phosphorylation is the phosphorylation and therefore the activation of myosin II, which in turn is needed for LE/Lys fission.

Our results further suggest that nexilin regulates myosin II activation through the interaction with the LE/Lys-associated calcium channel TRPML1. Indeed, myosin II phosphorylation by myosin II light chain kinase is calcium-dependent [41], and calcium release by TRPML1 promotes phosphorylation and activation of myosin II [3]. As nexilin has previously been described to regulate calcium dynamics by interacting with calcium channels in cardiac muscle cells [32], it is therefore not surprising that nexilin also affects the activity of the TRPML1 calcium channel. In line with this, nexilin depletion resulted in an accumulation of calcium in LE/Lys, and a reduction in TRPML1 presence on enlarged endosomes. Furthermore, the TRPML1 agonist ML-SA1 rescued the enlargement of the LE/Lys caused by nexilin knock down. This implies that TRPML1 activation was able to rescue the size of endosomes containing less TRPML1 channels. This may be due to smaller nearby TRPML1-positive endosomes that transiently fuse upon ML-SA1 addition with the enlarged endosomes to deliver TRPML1. In line with this hypothesis, it has been recently shown that local vesicle fusion is a prerequisite for TRPML1-mediated Ca^2+^ efflux [55]. This would facilitate calcium release followed by fission events as observed (Supplementary Video 2). It is also likely that calcium released from nearby endosomes trigger these fission events.

Our findings further suggest that nexilin is integral for the previously described interactions between Rab7b, TRPML1 and myosin II [36, 40], as nexilin depletion reduces the interaction between Rab7b and its interactors. In agreement with this, our microscopy data show that nexilin depletion resulted in a decreased presence of TRPML1 on enlarged Rab7b-positive LE/Lys. Intriguingly, both myosin II and TRPML1 depletion or inhibition have been reported to induce a similar LE/Lys enlargement [36, 42, 45], further supporting that these proteins work together to regulate LE/Lys dynamics.

The reduction in calcium release from TRPML1 upon nexilin knock down might affect calcium-dependent phosphorylation of myosin II, leading to deficient myosin II activity as previously reported [3]. In line with that, inhibition of myosin II activation is also observed upon Rab7b depletion [36, 40]. Hence, the LE/Lys enlargement observed upon nexilin knock down is likely a consequence of defective fission, as shown by live imaging of Rab7b-positive LE/Lys in nexilin depleted cells. Several lines of evidence support this model: fission is a calcium-regulated processes [56, 57], and TRPML1 activity has been shown to facilitate fission [42, 56]. Furthermore, myosin II activity regulates fission of early endosomes [27], mitochondria [58] and the Golgi [49]. Moreover, actin clusters accumulated on Rab7b-positive LE/Lys upon nexilin depletion (Fig. 5C-D), likely facilitated by the Arp2/3 complex [20], which has also been associated with deficiencies in the fission process [20]. Indeed, actin works together with myosin II to exert the membrane tension that is required for scission to occur [18, 21, 26, 27], and nexilin has previously been shown to promote actin polymerisation and plasticity [59]. The defective fission tubules observed on Rab7b-positive LE/Lys in cells depleted for nexilin, are likely due to a defect in the scission process, as has been previously reported [28, 60], further supporting this model.

**Fig. 6:**
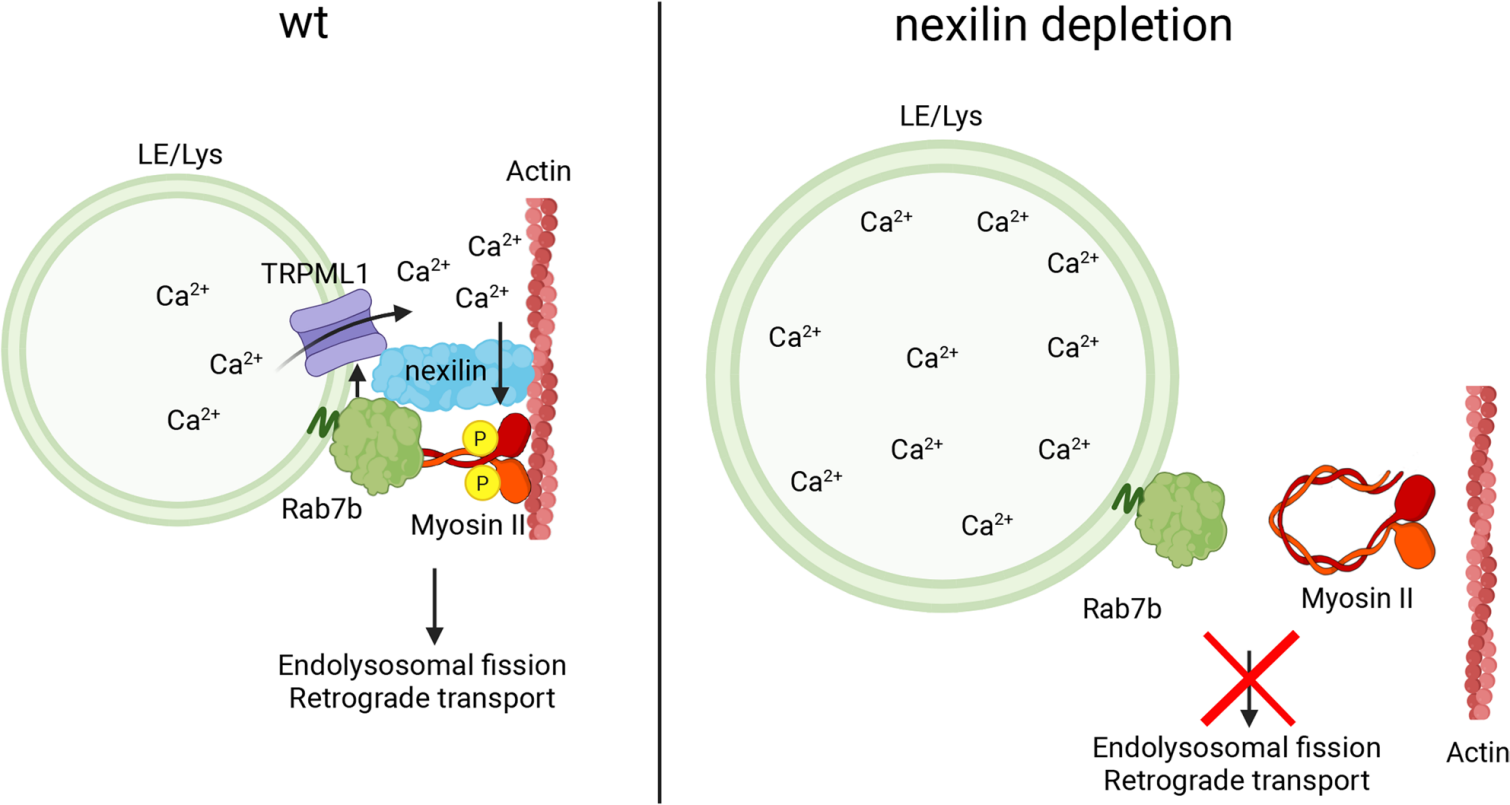
Model illustrating the role of nexilin in regulating calcium-dependent fission of LE/Lys. In wild-type (wt) cells, nexilin facilitates the interactions between Rab7b, myosin II, and the calcium channel TRPML1. Upon calcium release from the LE/Lys, myosin II is activated by calcium-dependent phosphorylation, triggering conformational change and binding to the actin cytoskeleton and Rab7b. This complex promotes fission of LE/Lys, important for retrograde transport to the Golgi. Upon nexilin depletion, the interactions of Rab7b with its interactors are reduced. Less TRPML1 channels are present on the membrane of LE/Lys, trapping calcium inside the LE/Lys. Myosin II phosphorylation is decreased due to reduced calcium release from LE/Lys and remains in its inactive conformation, unable to bind to Rab7b and the actin cytoskeleton. LE/Lys are thus unable to undergo fission, resulting in delayed retrograde transport. Created in BioRender. Bergundhaugen, M. (2025) https://BioRender.com/5ypqpx2

LE/Lys fission is crucial for protein sorting and delivery of cargo to the Golgi, a transport pathway mediated by Rab7b [37, 38]. In agreement with this, our results show that nexilin is required for Rab7b-mediated retrograde transport. Indeed, similarly to Rab7b depletion [37, 38], nexilin knock down resulted in delayed transport from LE/Lys to the Golgi, which was largely rescued by reintroducing nexilin. Intriguingly, TRPML1 depletion also leads to a similar delay in retrograde transport [61]. Together, these findings suggest that nexilin is required for retrograde transport from LE/Lys to the Golgi likely through calcium-regulated, myosin II-dependent fission at LE/Lys (Fig. 6).

In sum, our results identified nexilin as a novel regulator of LE/Lys fission and dynamics. We demonstrate that nexilin interacts with Rab7b, TRPML1 and myosin II. These interactions regulate lysosomal calcium release, and subsequent activation of myosin II to regulate LE/Lys fission events required for Rab7b-mediated retrograde transport.

## Methods

### Cell culture

U2OS and HeLa cells were cultured in Dulbecco’s Modified Eagle Medium (DMEM). PC3 cells were grown in Roswell Park Memorial Institute (RPMI) 1640 medium. Both mediums were supplemented with 10% fetal bovine serum (FBS, Biological Industries), 100 U/ml penicillin and 100 µg/ml streptomycin (Sigma-Aldrich). The cells grown at 5% CO_2_, 37 °C, and regularly tested for mycoplasma contamination.

### Antibodies and reagents

The following antibodies were used for immunofluorescence or western blot experiments: anti-Arp3 (ab49671, Abcam, 1:100), anti-GFP (ab6556, Abcam, 1:750), anti-GM130 (610822, BD Transduction Laboratories, 1:300), anti-Histidine (MCA1396, AbDserotec, 1:1000), anti-LAMP1 (H4A3, Developmental Studies Hybridoma Bank, University of Iowa, 1:1000), anti-myosin II (MYH9) (11128-1-AP, Proteintech, 1:2000), anti-myosin II light chain (M4401, Sigma-Aldrich, 1:200), anti-phosphorylated myosin II light chain (#3671, Cell Signaling Technology, 1:200), anti-nexilin (ab233235, Abcam, 1:2000–6000 for WB; HPA011904, Atlas Antibodies, 1:100 for IF), anti-Rab7a (2094 S, Cell Signaling Technology, 1:200), anti-Rab7b (H00338382-M01, AbNova, 1:50–200), anti-TRPML1 (ab272608, Abcam, 1:200), anti-tubulin (13–8000, Life Technologies, 1:100000). Secondary antibodies conjugated with horse peroxidase (HRP) (GE Healthcare, 1:5000) was used for western blotting. Secondary antibodies conjugated to Alexa Fluor 488, Alexa Fluor 555 or Alexa Fluor 647 (Life Technologies, 1:200) were used for immunofluorescence. DAPI (D9542, Sigma-Aldrich) was used at 0.1 μm/ml, Rhodamine-conjugated phalloidin (R415, Invitrogen) used at 33nM. ML-SA1 (SML0627, Sigma-Aldrich) was used for 1 h at 20 µM. LysoSensor yellow/blue DND-160 (Invitrogen) was used for 10 min at 2 µM before fixation. Cathepsin-B MR-(RR)2 Magic Red^®^ substrate (ImmunoChemistry technologies) was used according to the manufacturer’s instructions for 1 h before fixation.

### Constructs

The constructs pEGFP-C1-Rab7b, pCDNA3.1-mCherry-Rab7b, pET16b His-Rab7b and pET16b His-Rab9 have previously been described [37, 38]. pET16b His-Rab7a has been described [62]. Mucolipin1-pEGFP C3 was a gift from Paul Luzio (Addgene plasmid # 62960). pEGFP-C1-Rab5 from was a kind gift from Cecilia Bucci (University of Salento, Italy). pLAMP1-mCherry was a gift from Amy Palmer (Addgene plasmid # 45147), and pCMV-LifeAct-RFP was purchased from Ibidi. pCMV6-AC-GFP-nexilin was purchased from OriGene. siRNA-resistant nexilin-mTagBFP was purchased from GenScript. pCMV6-AC-GFP nexilin S442A and S442A were generated using the QuickChange II XL Site-Directed Mutagenesis Kits from Agilent Technologies, using manufacturer’s protocol, 18-minute extension time and 30 ng DNA. Nexilin S442A was first generated, the resulting plasmid was then used to generate the nexilin S442A mutant. The following primers were used: nexilin S442A sense 5’-ctgatcaaattaaaaaggagtggcgctattcaagctaaaaacctaaaaa-3’, antisense 5’-tttttaggtttttagcttgaatagcgccactcctttttaatttgatcag-3’; nexilin A442E sense 5’-ctgatcaaattaaaaaggagtggcgagattcaagctaaaaacctaaaaagca -3’, antisense 5’- tgctttttaggtttttagcttgaatctcgccactcctttttaatttgatcag-3’. Primers were designed using QuickChange Primer Design (agilent.com) and ordered from Eurofins Genomics.

### SiRNA screen

49 siRNA oligos from the human genome SMARTpool library (Horizon Discovery) were used (Table 1). PC3 cells were transfected with Lipofectamine RNAiMAX transfection reagent (Life Technologies) following the manufacturer’s protocol and 80 nM of siRNA. Briefly, siRNA oligos were added to a 96 well plate followed by addition of Lipofectamine RNAiMAX, and 4700 cells were added to the wells to a total volume of 100 µl. 30 µl were transferred to 18-well µ-slides (Ibidi, 81826), and incubated at 37 °C with 5% CO_2_ for 72 h. Cells in 18-well µ-slides were then fixed in 3% PFA for 15 min and permeabilized with 0.05% Saponin in PBS and immunostained. The wells were then covered with 80% glycerol in PBS. The samples were visualized using a Zeiss LSM880 Fast AiryScan confocal microscope equipped with 63 x oil Plan Apo NA 1.4 objective. Deconvolution of the pooled oligos was performed using the same protocol, but with each individual siRNA oligos (Dharmacon; Table 2) from the pool tested separately.

**Table 1:** SMARTpool SiRNAs (Dharmacon) used in the original screen

Gene symbol	Gene ID	siRNA Catalog Number
ON-TARGET plus Non-targeting Control	-	D-001810-10
PFN1	ENSG00000108518	L-012003-0
PFN2	ENSG00000070087	L-011750-00
MICAL2	ENSG00000133816	L-010189-00
FMN2	ENSG00000155816	L-022134-02
DIAPH1	ENSG00000131504	L-010347-00
SPIRE1	ENSG00000134278	L-023397-02
SPIRE2	ENSG00000204991	L-022605-02
RHOA	ENSG00000067560	L-003860-00
WDR1	ENSG00000071127	L-011984-00
TWF1	ENSG00000151239	L-003168-00
LIMK1	ENSG00000106683	L-007730-00
CFL1	ENSG00000172757	L-012707-00
CFL2	ENSG00000165410	L-019078-00
CDC42	ENSG00000070831	L-005057-00
RHOC	ENSG00000155366	L-008555-00
ROCK1	ENSG00000067900	L-003536-00
RAC1	ENSG00000136238	L-003560-00
RHOB	ENSG00000143878	L-008395-00
MICAL1	ENSG00000135596	L-010192-00
TRIOBP	ENSG00000100106	L-012342-00
MICAL3	ENSG00000243156	L-024432-00
PALLD	ENSG00000129116	L-016891-00
FSCN1	ENSG00000075618	L-019576-00
EPS8	ENSG00000151491	L-017905-00
WHAMM	ENSG00000156232	L-022415-01
TWF2	ENSG00000247596	L-003169-00
FSCN2	ENSG00000186765	L-020108-00
MPRIP	ENSG00000133030	L-014102-01
FLNB	ENSG00000136068	L-020175-00
FLNA	ENSG00000196924	L-012579-02
MICALL2	ENSG00000164877	L-014425-02
RHOD	ENSG00000173156	L-008940-00
TRIP10	ENSG00000125733	L-012685-00
LMOD2	ENSG00000170807	L-034869-02
CORO7	ENSG00000262246	L-014361-02
FAM21B	ENSG00000099290	L-026372-01
FAM21C	ENSG00000172661	L-029678-01
CORO1C	ENSG00000110880	L-017331-00
SCIN	ENSG00000006747	L-015062-00
ROCK2	ENSG00000134318	L-004610-00
PFN3	ENSG00000196570	L-024326-01
SHROOM3	ENSG00000138771	L-014123-00
NEXN	ENSG00000162614	L-016402-01
SVIL	ENSG00000197321	L-011398-00
RUFY3	ENSG00000018189	L-020336-00
RDX	ENSG00000137710	L-011762-00
AIF1	ENSG00000204472	L-011952-00
DMTN	ENSG00000158856	L-003663-02

**Table 2:** SiRNA oligos (Dharmacon) used in the Deconvolution screen

Gene Symbol	GENE ID	siRNA Catalog Number
MICAL2	ENSG00000133816	J-010189-05
MICAL2	ENSG00000133816	J-010189-06
MICAL2	ENSG00000133816	J-010189-07
MICAL2	ENSG00000133816	J-010189-08
SPIRE2	ENSG00000204991	J-022605-18
SPIRE2	ENSG00000204991	J-022605-19
SPIRE2	ENSG00000204991	J-022605-20
SPIRE2	ENSG00000204991	J-022605-21
CFL1	ENSG00000172757	J-012707-05
CFL1	ENSG00000172757	J-012707-06
CFL1	ENSG00000172757	J-012707-07
CFL1	ENSG00000172757	J-012707-08
FSCN2	ENSG00000186765	J-020108-05
FSCN2	ENSG00000186765	J-020108-06
FSCN2	ENSG00000186765	J-020108-07
FSCN2	ENSG00000186765	J-020108-08
MPRIP	ENSG00000133030	J-014102-09
MPRIP	ENSG00000133030	J-014102-10
MPRIP	ENSG00000133030	J-014102-11
MPRIP	ENSG00000133030	J-014102-12
FLNB	ENSG00000136068	J-020175-05
FLNB	ENSG00000136068	J-020175-06
FLNB	ENSG00000136068	J-020175-07
FLNB	ENSG00000136068	J-020175-08
FLNA	ENSG00000196924	J-012579-05
FLNA	ENSG00000196924	J-012579-06
FLNA	ENSG00000196924	J-012579-07
FLNA	ENSG00000196924	J-012579-08
CORO1C	ENSG00000110880	J-017331-05
CORO1C	ENSG00000110880	J-017331-06
CORO1C	ENSG00000110880	J-017331-07
CORO1C	ENSG00000110880	J-017331-08
NEXN	ENSG00000162614	J-016402-09
NEXN	ENSG00000162614	J-016402-10
NEXN	ENSG00000162614	J-016402-11
NEXN	ENSG00000162614	J-016402-12
ON-TARGETplus Non-targeting Control	-	D-001810-01

### Transfections and RNA interference

U2OS and HeLa cells were transfected at ~ 70% confluency using Lipofectamine 2000 (Invitrogen), and PC3 cells using FuGENE 6 (ProMega), overnight, according to manufacturer’s protocol. For transfection with GFP-nexilin plasmids, U2OS cells were electroporated using the Amaxa Nucleofector II/2b system according to manufacturer’s protocol, using the X-001 program. For transfection with siRNAs, cells were plated the day prior transfection, and transfected at ~ 20% confluency using RNAiMAX Transfection Reagent (Life Technologies) according to manufacturer’s protocol. The experiments were performed 72 hours after transfection. The following siRNA oligos were used: Nexilin siRNA#1 sense 5’-GGAGAUGAUUCACUACU-3’, antisense 5’-AGUAGUGAAUCAUCUCC-3’; nexilin siRNA #2 sense 5’-AUAUGGUAGUAGAUGA-3’, antisense 5’-AUCAUCUACUACCAUAU-3’; Rab7a siRNA sense 5’-GGAUGACCUCUAGGAAGAA-3′ and antisense 5′-UUCUUCCUAGAGGUCAUCC-3′. As negative control, we used the sense 5’-ACUUCGAGCGCGUGCAUGGC-3’, antisense 5’-AGCCAUGCACGCUCGAAGU-3’. The oligonucleotides were purchased from Eurofins Genomics.

### Co-immunoprecipitation and pulldown experiments

GFP-TRAP^®^_MA for immunoprecipitation of GFP fusion proteins (Chromotek) was used according to manufacturer’s protocol. Cells were transfected, then lysed 24 h after transfection, washed and incubated with magnetic agarose beads for 1 h in end-over-end rotation. Binding Control Agarose Beads (Chromotek) and samples transfected with a control vector (pEGFP-C1) were used as negative controls. Immunoprecipitated samples were loaded on SDS-PAGE gels and subjected with western blot analysis.

His-tagged Rabs were expressed in Escherichia coli BL21 (DE3) (Agilent Technologies) upon induction with 0.5 mM IPTG for 3 h at 37 °C. His-tag isolation Dynabeads (Invitrogen) were used to purify His-Rabs from the bacterial soluble fractions with 50 mM Na-phosphate pH 8, 300 mM NaCl and 0.01% Tween 20, according to the manufacturer’s protocol. The purified His-tagged Rabs were activated by loading them with 0.1 mM GTPγS. For pull-down experiments, His-tag isolation Dynabeads were loaded with 20 µg of GTPγS-His-Rab fusion proteins and incubated with precleared HeLa cell lysate for 30 min at 4 °C. After six washes with a buffer containing 3.25 mM Na-phosphate, pH 7.4, 70 mM NaCl and 0.01% Tween 20, the bound proteins were eluted with elution buffer (50 mM Na-phosphate pH 8.0, 300 mM NaCl, 0.01% Tween 20, and 300 mM Imidazole).

### SDS-PAGE and Western blotting

For western blot analysis, proteins were separated by SDS-PAGE, transferred to a polyvinylidene fluoride (PVDF) membrane (Millipore), and subjected to immunoblot analysis by overnight incubation at 4 °C with primary antibodies diluted in 2% blotting-grade non-fat dry milk (BioRad), followed by washing and incubation for one hour at room temperature with HRP-conjugated secondary antibodies. The protein bands were visualised using the ECL system (GE Healthcare) or SuperSignal solution (Thermo Scientific). Band intensities were quantified through densitometry analysis using ImageJ.

### Immunofluorescence and live-imaging microscopy

Cells grown on coverslips were fixed with 3% PFA at room temperature for 20 min, washed with PBS 1X, quenched for 10 min with 50mM NH_4_Cl, permeabilised for 10 min with 0.25% saponin in PBS 1X before staining. For staining with the Arp3 antibody, the cells were permeabilised for 15 min in 0.1% Triton X-100 (Sigma-Aldrich) in PBS 1X prior to staining. The samples were incubated with primary antibodies for 20 min at room temperature. The cells were washed and incubated with appropriate secondary antibodies for 20 min at room temperature before washing and mounting on glass slides using mowiol. For live imaging experiments, cells were grown on MatTek glass-bottom dishes. The cells were imaged using a Zeiss LSM880 Fast AiryScan confocal microscope equipped with 63 x oil Plan Apo NA 1.4 objective for super-resolution imaging of fixed samples or an Olympus SpinSR10 spinning disk confocal super resolution microscope using a 60x oil Planpon NA 1.42 objective for super-resolution or confocal imaging of fixed samples or for live-cell imaging at 37 °C, 5% CO_2_.

### Calcium imaging

U2OS cells were grown on MatTek glass-bottom dishes one day before transfection with siRNA against nexilin and incubated for 48 h at 5% CO_2_, 37 °C before transfection with mCherry-tagged Rab7b. After 24 h, the cells were incubated with 2µM Fluo-4 AM in Hanks’ Balanced Salt Solution (HBSS), supplemented with 2% FBS for 30 min. The cells were washed with HBSS four times before live-cell imaging. To determine the ratio of Fluo-4 AM in LE/Lys against the cytosol Fluo-4 AM-positive puncta were thresholded and their mean intensity was measured using ImageJ and compared to the Fluo-4 AM total mean intensity in the cell. For measurements of Fluo-4 AM signal in LE/Lys, raw integrated density was measured from the Fluo-4 AM puncta using ImageJ and normalised to the control condition; measurements of Fluo-4 AM in the cytosol was determined by measuring the total raw integrated density in the cell, subtracting the raw integrated density of the Fluo-4 AM in LE/Lys, and normalised to the control condition.

### Cholera-toxin uptake

Cholera-toxin uptake was performed as previously described [37]. U2OS cells grown on coverslips were incubated with 4 μm/ml Cholera-toxin subunit B (Recombinant) Alexa Fluor 555 conjugate (Invitrogen) for 45 min (Fig. 5A-B) or 1 h (Supplementary Fig. 5A-B) at 5% CO_2_, 37 °C. The cells were washed and chased for 30 min at 5% CO_2_, 37 °C before fixation using 3% PFA and immunostaining.

### Late endosomal fission measurements

U2OS cells were imaged with the Olympus SpinSR10 spinning disk confocal super resolution microscope. For imaging of fission events shown in Fig. 5E-G, images were acquired every 10 s for 15 min. For imaging of fission events shown in Supplementary Fig. 5E-F, Supplementary Fig. 6 and Supplementary Video S1, the cells were imaged in stacks of 3–5 z-planes every 5 min for 15 h.

Fission tubules were analysed manually in ImageJ for both experiments by marking each fission tubule in every frame of the acquired movies, making sure the same tubule was not counted twice. The length of the fission tubule was measured by manually marking each tubule with the segmented line tool in ImageJ, and was only measured in the frame where the tubule reached its maximum length.

### Image processing, analysis and statistical analysis

Images were processed and analysed using ImageJ. Vesicle size was analysed using the CellPose plug-in in ImageJ [63]. Co-localisation was analysed by obtaining Mander’s coefficient using the JACoP plug-in in ImageJ [64]. Statistical analysis was done using two-tailed unpaired Student’s *t*-test or a one-way or two-way Analysis of Variance (ANOVA) followed by a Tukey’s or Šidák post-hoc test in Prism 10/GraphPad, depending on if two or more samples were compared to each other, respectively. Statistical significance is indicated as follows: ns non-significant, *p < 0.05, **p < 0.01, ***p < 0.001, ****p < 0.0001. Results are expressed as mean ± s.d.

## Supplementary Information

Supplementary Material 1: Figure S1. Nexilin does not affect lysosomal function or early endosome size. **A** Quantification of nexilin protein levels in PC3 cells transfected with control siRNA or six different siRNAs against nexilin from Western blotting, quantified using densitometry using ImageJ. The protein levels were normalized to tubulin, and plotted relative to the control. Data represents the mean ± s.d. from three independent experiments, two-tailed unpaired Student’s t-test was applied for statistical analysis. **B** U2OS cells were transfected with control siRNA, siRNAs targeting nexilin, or siRNA against Rab7a, and incubated with Magic Red for 1 hour before fixation and imaging. Scale bar: 10 µm. **C** Quantification of the average mean intensity of Magic Red per cell, relative to the control. Data represents the mean ± s.d. from 3 independent experiments; 2 experiments for siRab7a (n = 60 cells in total per condition, n = 40 cells in total for siRab7a), two-tailed, unpaired Student’s t-test was applied for statistical analysis. **D** U2OS cells transfected with control siRNA, siRNAs against nexilin or siRNA against Rab7a were lysed and subjected to Western blot analysis using antibodies against nexilin and Rab7a, and tubulin as loading control. **E** U2OS cells were transfected with siRNAs against nexilin or non-targeting control siRNA, and transiently transfected with GFP-Rab5 to label early endosomes before live cell imaging. Scale bar: 10 µm. **F** Quantification of early endosome size (µm²) per cell in U2OS cells transfected with control siRNA or siRNAs targeting nexilin. Data represents the mean ± s.d. for three independent experiments (n = 45 cells in total per condition), two-tailed unpaired Student’s t-test was applied for analysis. * p < 0.05, ** p < 0.01, *** p < 0.001, **** p < 0.0001, ns non-significant

Supplementary Material 2: Figure S2. Nexilin regulates the size of Rab7b-positive LE/Lys in different cell lines. **A** U2OS, PC3 and HeLa cells were lysed and subjected to Western blot analysis using antibodies against nexilin, and against tubulin as loading control. **B** PC3 cells were transfected with siRNAs against nexilin or non-targeting control siRNA, and transiently transfected for GFP-Rab7b before live cell imaging. Scale bar: 10 μm. **C** Quantification of the number of large LE/Lys with an area over 3 μm per cell after nexilin knock down. Data represents the mean ± s.d. for 2 independent experiments (n = 30 cells in total per condition). **D** HeLa cells were transfected with siRNAs against nexilin or non-targeting control siRNA and transiently transfected with GFP-Rab7b before live cell imaging. Scale bar: 10 μm. **E** HeLa cells transfected with siRNA against nexilin or non-targeting siRNA were lysed and subjected to Western blot analysis using antibodies against nexilin and tubulin. **F** Amino acid sequences of nexilin. The phosphorylation site (S) and the corresponding mutated amino acids are indicated in red. **G** Quantification of colocalisation of Rab7b with nexilin from U2OS cells co-transfected with mCherry-Rab7b and GFP-Nexilin wild type (wt), S442E (phosphorylation-mimic mutant) or S442A (phosphorylation-null mutant), and imaged using a super resolution spinning disk microscope. Data represents the mean ± s.d. from 3 independent experiments (n ≥ 19 cells in total per condition), one-way ANOVA followed by Tukey’s post-hoc test was applied for statistical analysis

Supplementary Material 3: Figure S3. Phosphorylation of nexilin alters myosin II phosphorylation. **A** U2OS cells transiently transfected with GFP-nexilin wild type (wt), S442E (phosphorylation-mimic mutant), or S442A (phosphorylation-null mutant) were lysed and subjected to Western blot analysis using antibodies against phosphorylated myosin light chain on Ser 19 (pMLC), total myosin light chain (MLC), nexilin and tubulin. **B** Quantification of the levels of phosphorylated myosin light chain on Ser 19 (pMLC). The intensity of the bands from the Western blots were measured using ImageJ and normalised to the total myosin light chain (MLC) levels, using tubulin as loading control. Data represents the mean ± s.d. from three independent experiments. One-way ANOVA followed by Tukey’s post-hoc test was applied for statistical analysis. **C** U2OS cells transiently transfected with mCherry-LAMP1 were treated with 20µM ML-SA1 for 1 hour, or left untreated, then incubated with 2µM LysoSensor Yellow/Blue DND-160 for 10 minutes before fixation and imaging Scale bar: 10 µm. ns non-significant, * p < 0.05, ** p < 0.01

Supplementary Material 4: Figure S4. Calcium accumulation in LE/Lys is rescued by reintroduction of nexilin. **A** U2OS cells treated with control siRNAs, siRNAs against nexilin, or siRNAs against nexilin and then transfected with siRNA-resistant BFP-nexilin, were transfected with mCherry-Rab7b and incubated with Fluo-4 AM for 30 minutes before live cell imaging. Scale bar: 10 µm. **B** Intensity map showing Fluo-4 AM intensity for the cells shown in A). Scale bar: 10 µm. **C** Average mean intensity of Fluo-4 AM fluorescence in LE/Lys over the cytosol. Data represents mean ± s.d. from three independent experiments (n = 60 cells in total per condition). One-way ANOVA followed by a Tukey’s post hoc test was applied for statistical analysis. ns non-significant, ** p < 0.01

Supplementary Material 5: Figure S5. Nexilin depletion alters cholera toxin B retrograde transport to the Golgi and prevents LE/Lys fission. **A** U2OS cells were transfected with control siRNA, siRNAs against nexilin, and incubated with 4 mg/ml cholera toxin subunit B conjugated with AlexaFluor-555 (green) for 1 hour, then chased for 30 minutes before fixation and staining using a primary antibody against GM130 (magenta). White squares indicate magnified areas. Scale bar: 5 µm. **B** Quantification of colocalization of CTxB with GM130 using ImageJ to obtain Mander’s coefficient. Data represents mean ± s.d. from three independent experiments (n = 60 cells in total per condition). Two-tailed, unpaired Student’s t-test was applied for statistical analysis. **C** U2OS cells treated with control siRNA or siRNAs targeting nexilin were transiently transfected with GFP-Rab7b (green) before fixation and staining using rhodamine phalloidin (grey) and antibodies against Arp3 (magenta). White squares indicate magnified areas. Scale bar: 5 µm. **D** Quantification of the number of Arp3-positive actin clusters on Rab7b-positive endosomes per cell. Data represents mean ± s.d. from three independent experiments (n ≥ 41 cells in total per condition). Two-tailed, unpaired Student’s t-test was applied for statistical analysis. **E** U2OS cells were treated with control siRNA or siRNAs against nexilin, and transiently transfected with GFP-Rab7b before live cell imaging. White squares indicate the magnified area. Arrows point to fission tubules. Scale bar: 5 µm. **F** The graph represents the average number of Rab7b-positive fission tubules detected per hour per cell over 15 hours of live-cell imaging (n ≥ 9 cells in total per condition). Two-tailed, unpaired Student’s t-test was applied for statistical analysis. * p < 0.05, ** p < 0.01, *** p < 0.001

Supplementary Material 6: Figure S6. Rab7b-positive LE/Lys dynamics over time in cells knocked down for nexilin. **A** U2OS cells were treated with control siRNA or siRNAs against nexilin, and transiently transfected with GFP-Rab7b before live cell imaging over 15 hours, starting 6 hours after transfection. Scale bar: 5 µm

Supplementary Material 7: Video S1. Rab7b-positive LE/Lys dynamics over time in cells knocked down for nexilin. U2OS cells were transfected with control siRNA or siRNAs targeting nexilin, and transiently transfected with GFP-Rab7b. The cells were imaged 6 hours after transfection with GFP-Rab7b, and imaged every 5 minutes for 15 hours. Scale bar: 5 µm

Supplementary Material 8: Video S2. Calcium is released from LE/Lys upon incubation with ML-SA1. U2OS cells depleted for nexilin were loaded with Fluo-4 AM for 30 minutes. 20 µM ML-SA1 was added to the cells before live cell imaging every 2 minutes for 1 hour using a spinning disk confocal microscope. Scale bar: 5 µm

Supplementary Material 9

## Data Availability

No datasets were generated or analysed during the current study. Data is provided within the manuscript or supplementary information files.
