# Supplementary figures and images for: Nexilin promotes calcium-dependent endo-lysosomal fission required for retrograde transport
Source: Cell Commun Signal. 2026 Jan 9;24:110. doi: 10.1186/s12964-025-02628-8 (PMC12896057; doi:10.1186/s12964-025-02628-8)

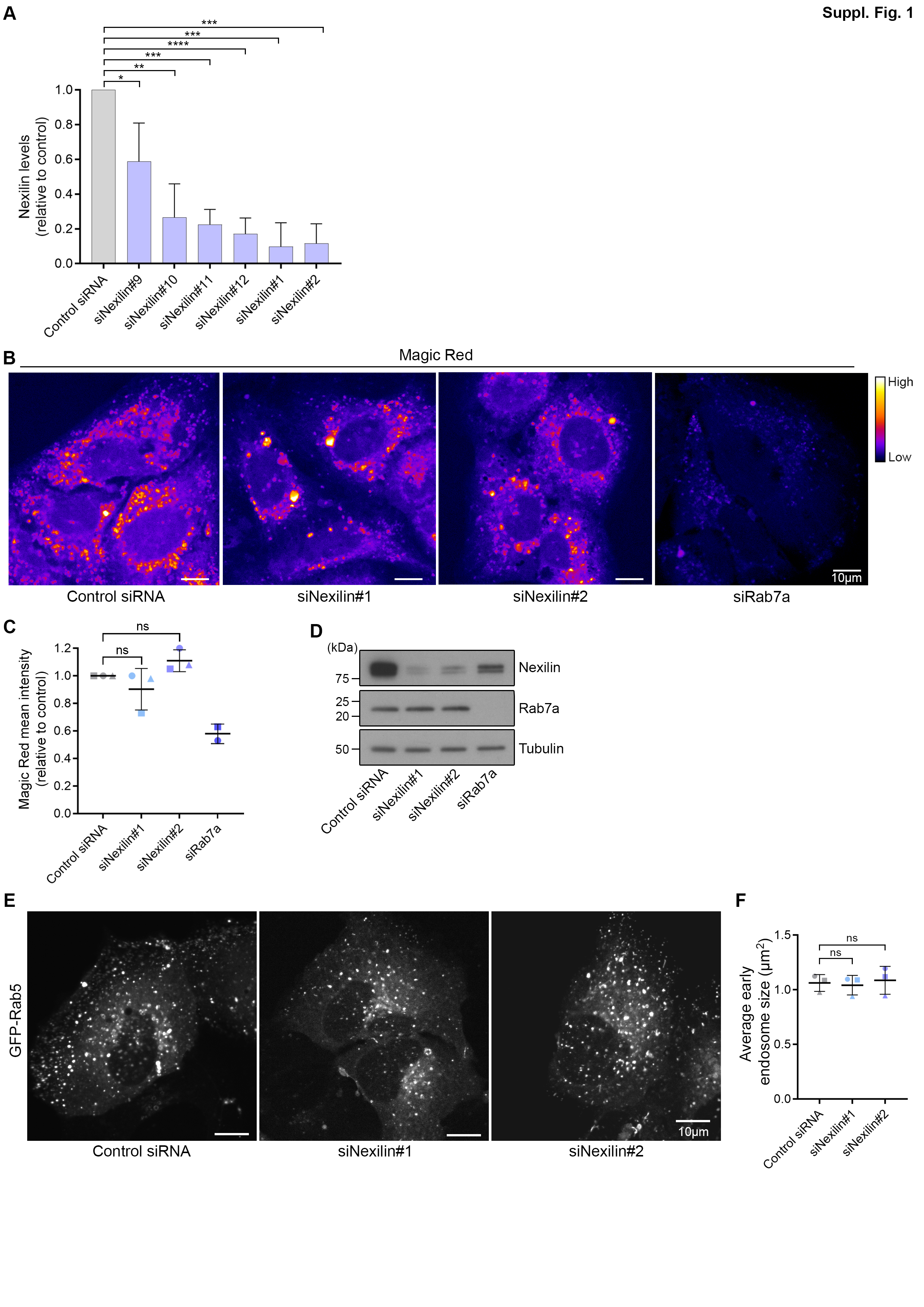

Supplement: Supplementary file 1 — Supplementary Material 1: Figure S1. Nexilin does not affect lysosomal function or early endosome size. A Quantification of nexilin protein levels in PC3 cells transfected with control siRNA or six different siRNAs against nexilin from Western blotting, quantified using densitometry using ImageJ. The protein levels were normalized to tubulin, and plotted relative to the control. Data represents the mean ± s.d. from three independent experiments, two-tailed unpaired Student’s t-test was applied for statistical analysis. B U2OS cells were transfected with control siRNA, siRNAs targeting nexilin, or siRNA against Rab7a, and incubated with Magic Red for 1 hour before fixation and imaging. Scale bar: 10 µm. C Quantification of the average mean intensity of Magic Red per cell, relative to the control. Data represents the mean ± s.d. from 3 independent experiments; 2 experiments for siRab7a (n = 60 cells in total per condition, n = 40 cells in total for siRab7a), two-tailed, unpaired Student’s t-test was applied for statistical analysis. D U2OS cells transfected with control siRNA, siRNAs against nexilin or siRNA against Rab7a were lysed and subjected to Western blot analysis using antibodies against nexilin and Rab7a, and tubulin as loading control. E U2OS cells were transfected with siRNAs against nexilin or non-targeting control siRNA, and transiently transfected with GFP-Rab5 to label early endosomes before live cell imaging. Scale bar: 10 µm. F Quantification of early endosome size (µm²) per cell in U2OS cells transfected with control siRNA or siRNAs targeting nexilin. Data represents the mean ± s.d. for three independent experiments (n = 45 cells in total per condition), two-tailed unpaired Student’s t-test was applied for analysis. * p < 0.05, ** p < 0.01, *** p < 0.001, **** p < 0.0001, ns non-significant [file 12964_2025_2628_MOESM1_ESM.tif]

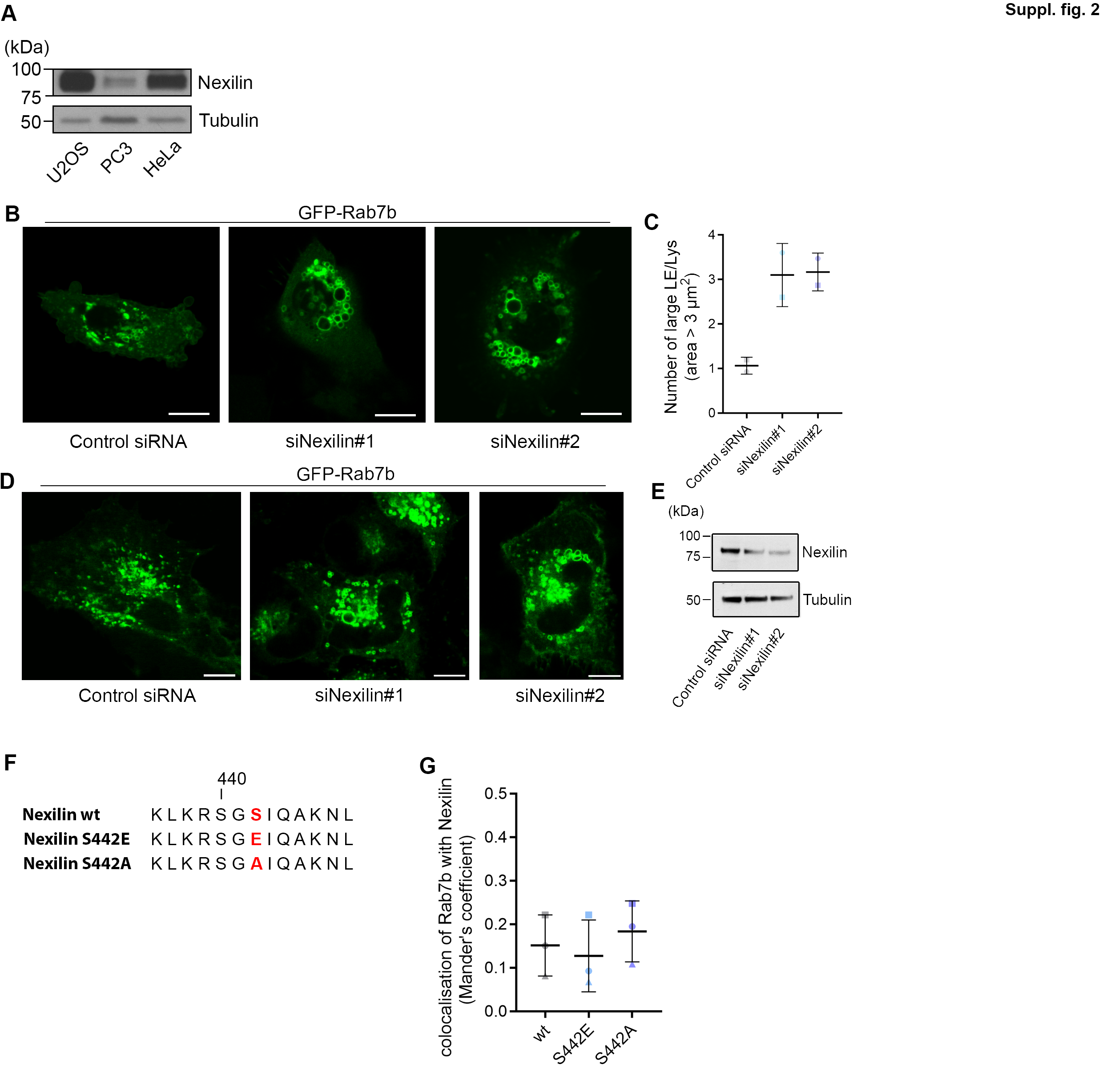

Supplement: Supplementary file 2 — Supplementary Material 2: Figure S2. Nexilin regulates the size of Rab7b-positive LE/Lys in different cell lines. A U2OS, PC3 and HeLa cells were lysed and subjected to Western blot analysis using antibodies against nexilin, and against tubulin as loading control. B PC3 cells were transfected with siRNAs against nexilin or non-targeting control siRNA, and transiently transfected for GFP-Rab7b before live cell imaging. Scale bar: 10 μm. C Quantification of the number of large LE/Lys with an area over 3 μm per cell after nexilin knock down. Data represents the mean ± s.d. for 2 independent experiments (n = 30 cells in total per condition). D HeLa cells were transfected with siRNAs against nexilin or non-targeting control siRNA and transiently transfected with GFP-Rab7b before live cell imaging. Scale bar: 10 μm. E HeLa cells transfected with siRNA against nexilin or non-targeting siRNA were lysed and subjected to Western blot analysis using antibodies against nexilin and tubulin. F Amino acid sequences of nexilin. The phosphorylation site (S) and the corresponding mutated amino acids are indicated in red. G Quantification of colocalisation of Rab7b with nexilin from U2OS cells co-transfected with mCherry-Rab7b and GFP-Nexilin wild type (wt), S442E (phosphorylation-mimic mutant) or S442A (phosphorylation-null mutant), and imaged using a super resolution spinning disk microscope. Data represents the mean ± s.d. from 3 independent experiments (n ≥ 19 cells in total per condition), one-way ANOVA followed by Tukey’s post-hoc test was applied for statistical analysis [file 12964_2025_2628_MOESM2_ESM.tif]

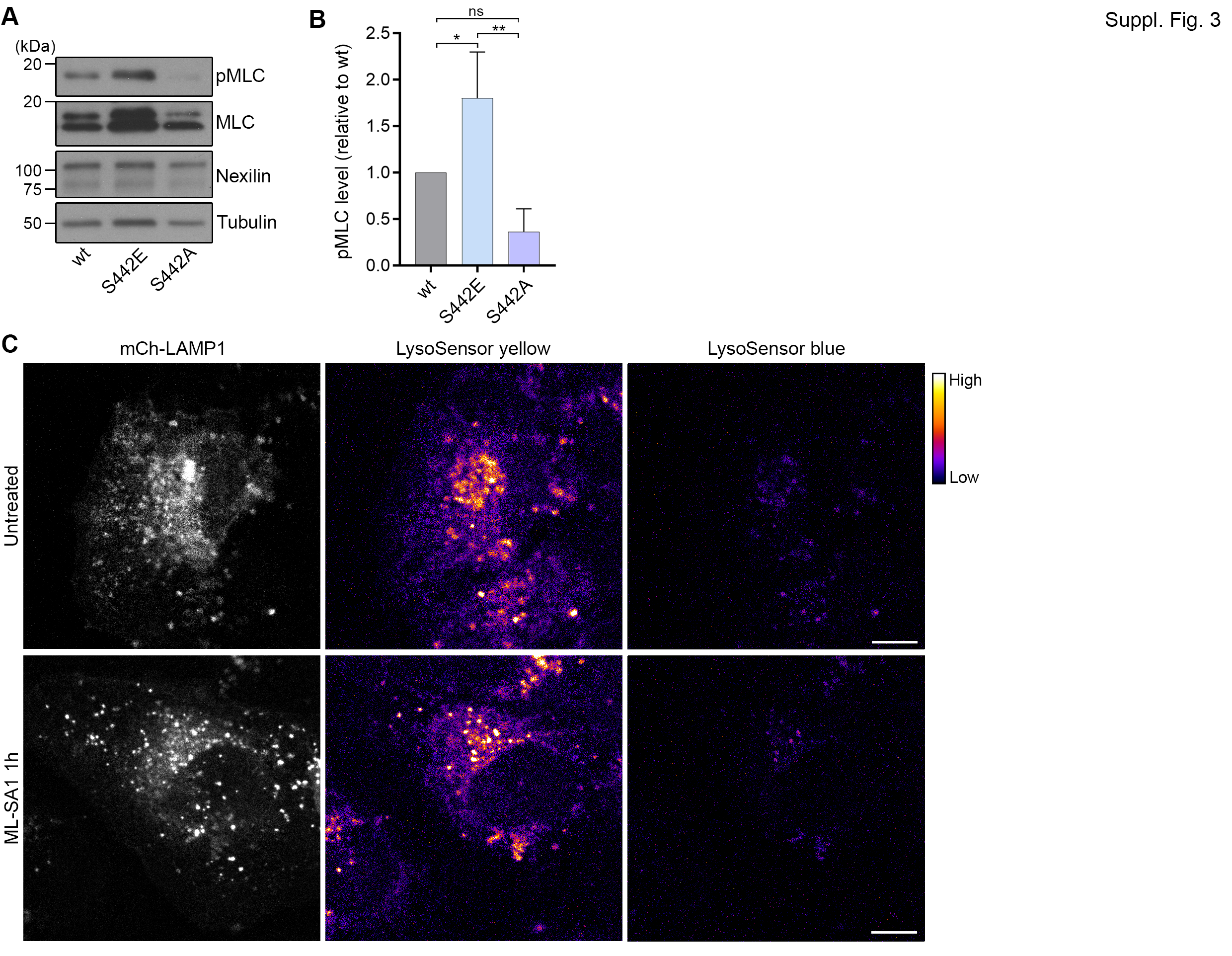

Supplement: Supplementary file 3 — Supplementary Material 3: Figure S3. Phosphorylation of nexilin alters myosin II phosphorylation. A U2OS cells transiently transfected with GFP-nexilin wild type (wt), S442E (phosphorylation-mimic mutant), or S442A (phosphorylation-null mutant) were lysed and subjected to Western blot analysis using antibodies against phosphorylated myosin light chain on Ser 19 (pMLC), total myosin light chain (MLC), nexilin and tubulin. B Quantification of the levels of phosphorylated myosin light chain on Ser 19 (pMLC). The intensity of the bands from the Western blots were measured using ImageJ and normalised to the total myosin light chain (MLC) levels, using tubulin as loading control. Data represents the mean ± s.d. from three independent experiments. One-way ANOVA followed by Tukey’s post-hoc test was applied for statistical analysis. C U2OS cells transiently transfected with mCherry-LAMP1 were treated with 20µM ML-SA1 for 1 hour, or left untreated, then incubated with 2µM LysoSensor Yellow/Blue DND-160 for 10 minutes before fixation and imaging Scale bar: 10 µm. ns non-significant, * p < 0.05, ** p < 0.01 [file 12964_2025_2628_MOESM3_ESM.tif]

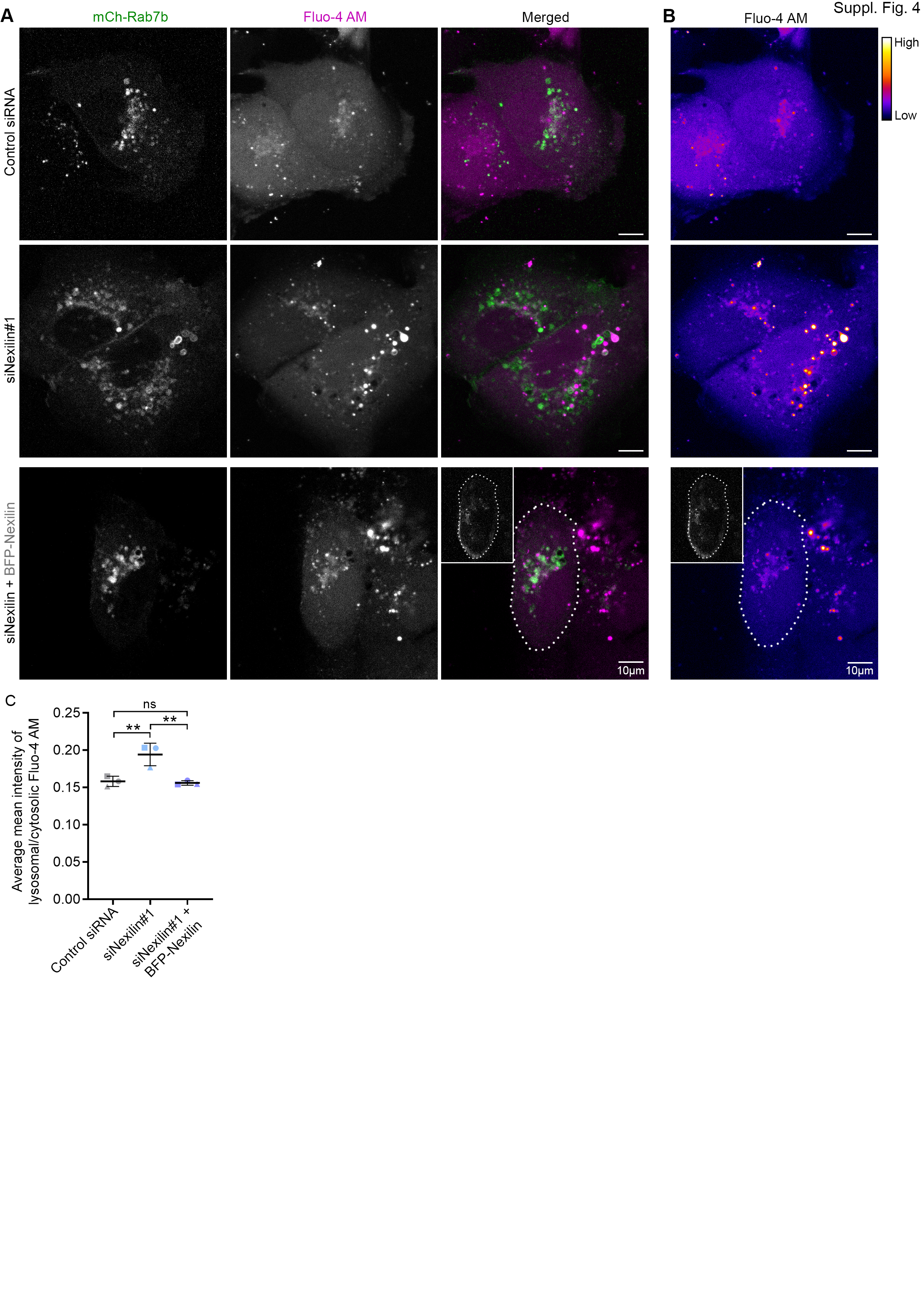

Supplement: Supplementary file 4 — Supplementary Material 4: Figure S4. Calcium accumulation in LE/Lys is rescued by reintroduction of nexilin. A U2OS cells treated with control siRNAs, siRNAs against nexilin, or siRNAs against nexilin and then transfected with siRNA-resistant BFP-nexilin, were transfected with mCherry-Rab7b and incubated with Fluo-4 AM for 30 minutes before live cell imaging. Scale bar: 10 µm. B Intensity map showing Fluo-4 AM intensity for the cells shown in A). Scale bar: 10 µm. C Average mean intensity of Fluo-4 AM fluorescence in LE/Lys over the cytosol. Data represents mean ± s.d. from three independent experiments (n = 60 cells in total per condition). One-way ANOVA followed by a Tukey’s post hoc test was applied for statistical analysis. ns non-significant, ** p < 0.01 [file 12964_2025_2628_MOESM4_ESM.tif]

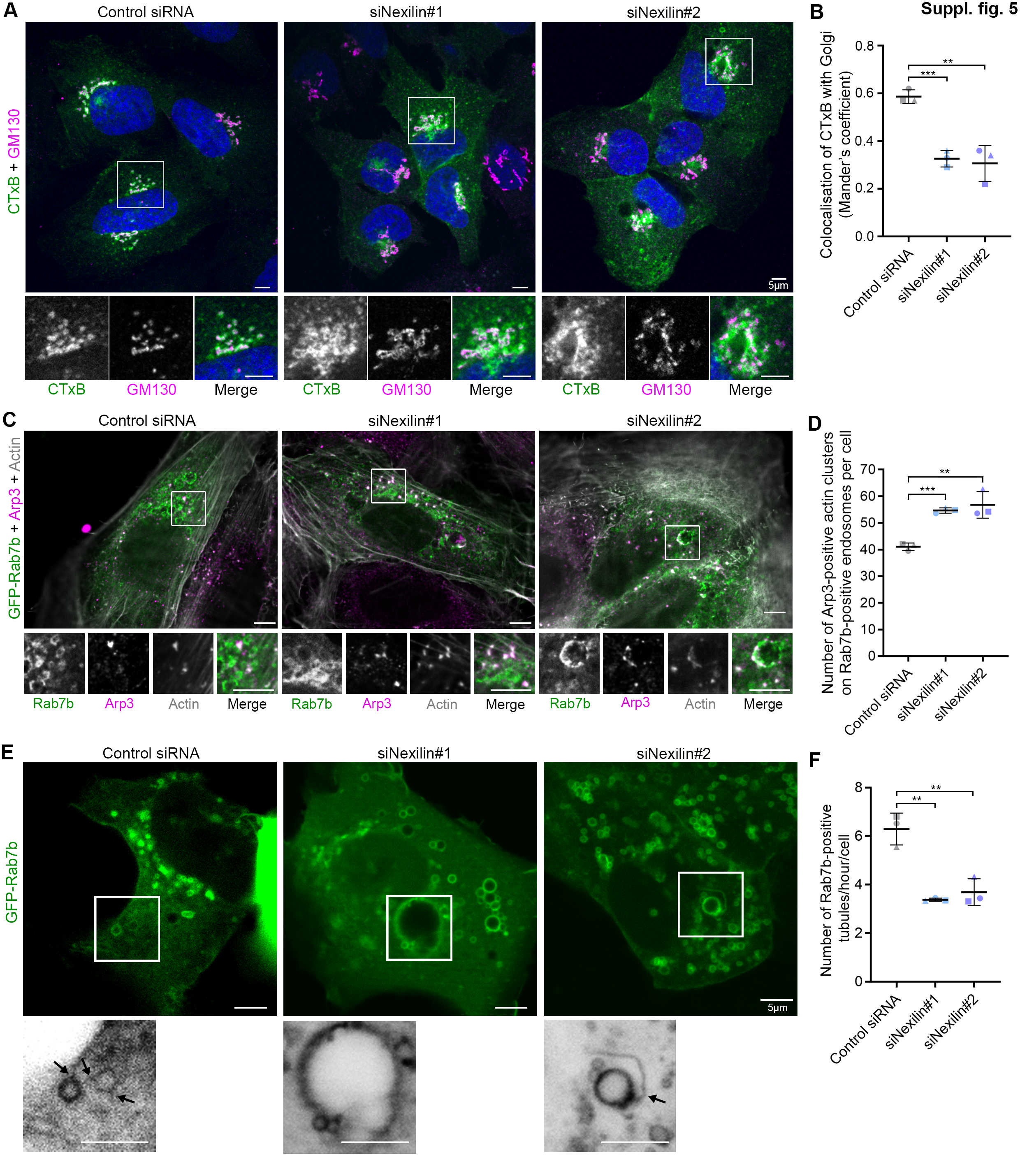

Supplement: Supplementary file 5 — Supplementary Material 5: Figure S5. Nexilin depletion alters cholera toxin B retrograde transport to the Golgi and prevents LE/Lys fission. A U2OS cells were transfected with control siRNA, siRNAs against nexilin, and incubated with 4 mg/ml cholera toxin subunit B conjugated with AlexaFluor-555 (green) for 1 hour, then chased for 30 minutes before fixation and staining using a primary antibody against GM130 (magenta). White squares indicate magnified areas. Scale bar: 5 µm. B Quantification of colocalization of CTxB with GM130 using ImageJ to obtain Mander’s coefficient. Data represents mean ± s.d. from three independent experiments (n = 60 cells in total per condition). Two-tailed, unpaired Student’s t-test was applied for statistical analysis. C U2OS cells treated with control siRNA or siRNAs targeting nexilin were transiently transfected with GFP-Rab7b (green) before fixation and staining using rhodamine phalloidin (grey) and antibodies against Arp3 (magenta). White squares indicate magnified areas. Scale bar: 5 µm. D Quantification of the number of Arp3-positive actin clusters on Rab7b-positive endosomes per cell. Data represents mean ± s.d. from three independent experiments (n ≥ 41 cells in total per condition). Two-tailed, unpaired Student’s t-test was applied for statistical analysis. E U2OS cells were treated with control siRNA or siRNAs against nexilin, and transiently transfected with GFP-Rab7b before live cell imaging. White squares indicate the magnified area. Arrows point to fission tubules. Scale bar: 5 µm. F The graph represents the average number of Rab7b-positive fission tubules detected per hour per cell over 15 hours of live-cell imaging (n ≥ 9 cells in total per condition). Two-tailed, unpaired Student’s t-test was applied for statistical analysis. * p < 0.05, ** p < 0.01, *** p < 0.001 [file 12964_2025_2628_MOESM5_ESM.tif]

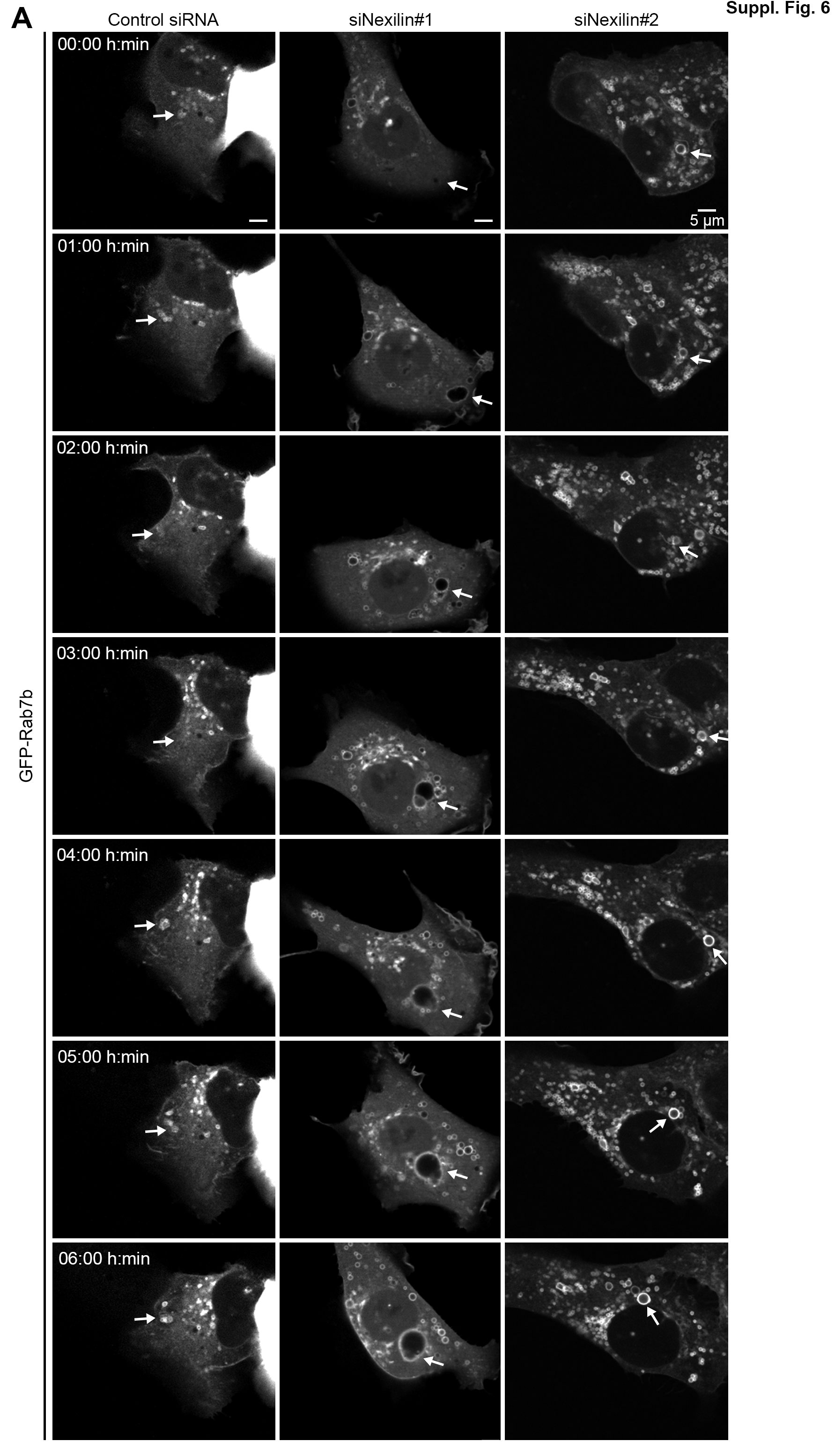

Supplement: Supplementary file 6 — Supplementary Material 6: Figure S6. Rab7b-positive LE/Lys dynamics over time in cells knocked down for nexilin. A U2OS cells were treated with control siRNA or siRNAs against nexilin, and transiently transfected with GFP-Rab7b before live cell imaging over 15 hours, starting 6 hours after transfection. Scale bar: 5 µm [file 12964_2025_2628_MOESM6_ESM.tif]
