# Supplementary material for: Nexilin promotes calcium-dependent endo-lysosomal fission required for retrograde transport
Source: Cell Commun Signal. 2026 Jan 9;24:110. doi: 10.1186/s12964-025-02628-8 (PMC12896057; doi:10.1186/s12964-025-02628-8)

Figure 1

C

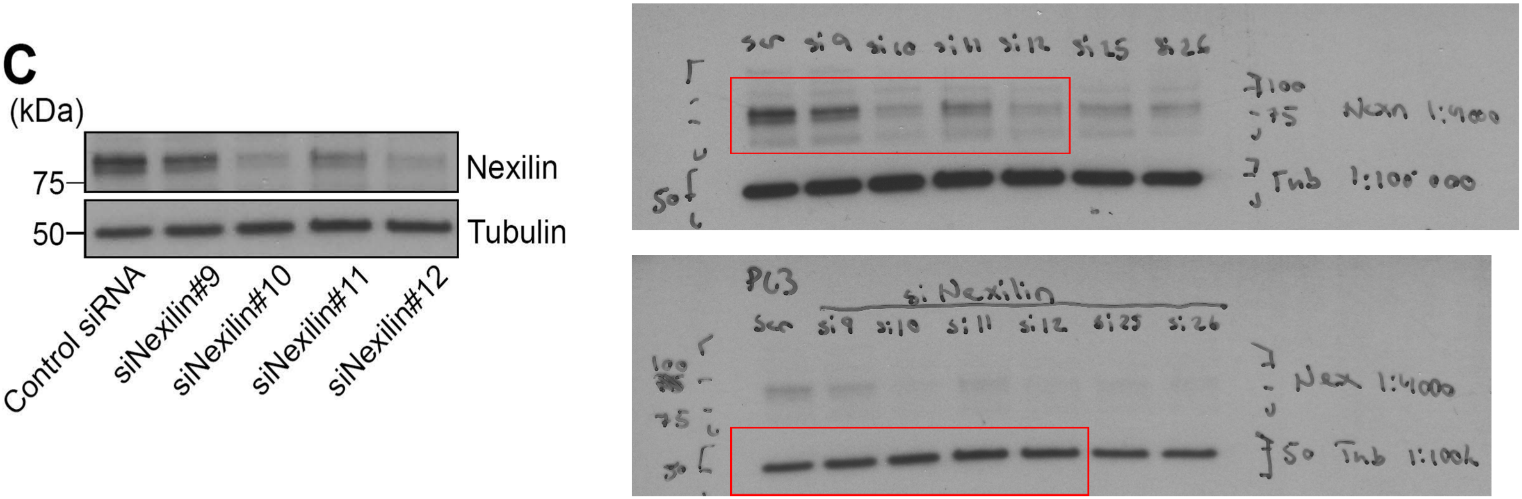

(kDa)
75
50
Nexilin
Tubulin
Control siRNA
siNexilin#9
siNexilin#10
siNexilin#11
siNexilin#12


E

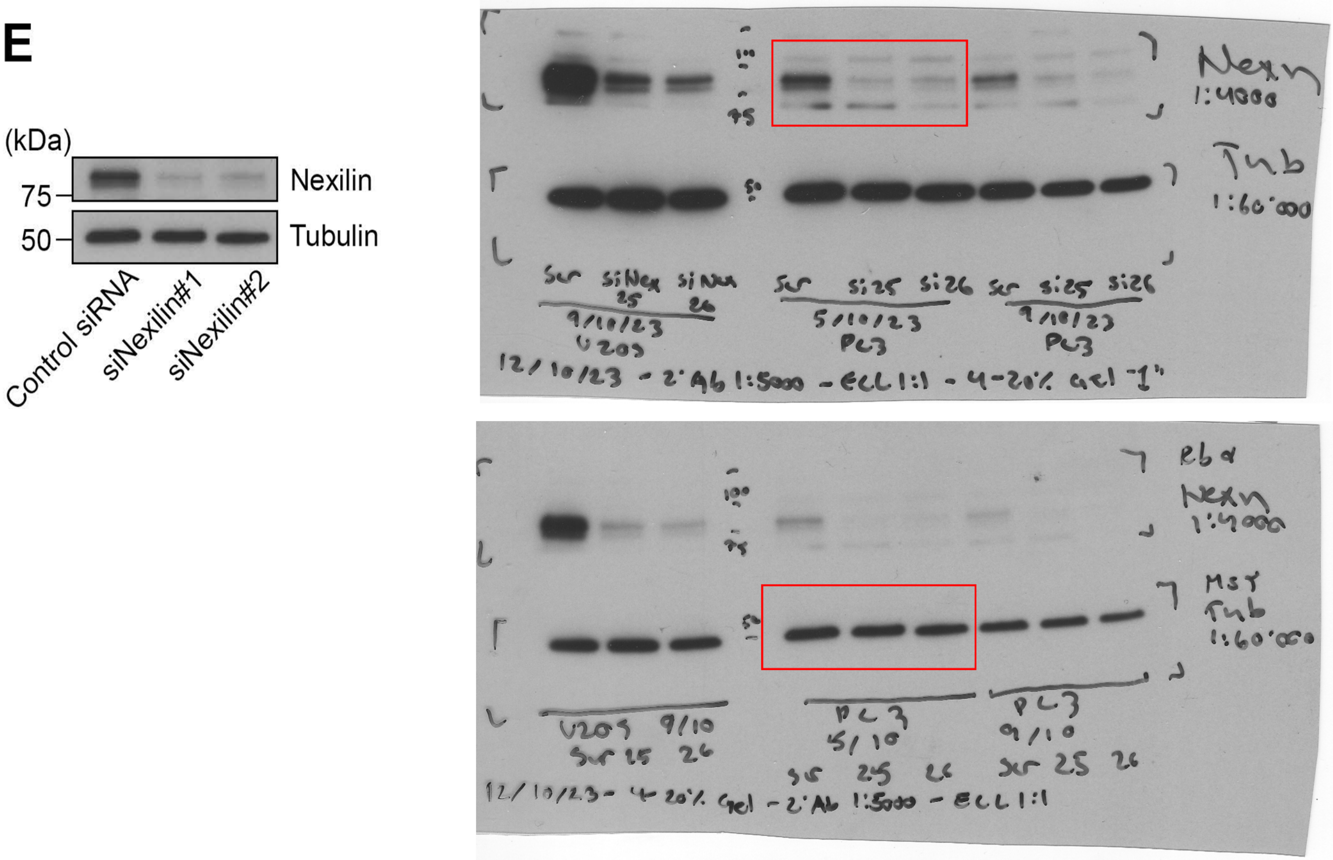

(kDa)
75
50
Nexilin
Tubulin
Control siRNA
siNexilin#1
siNexilin#2

Figure 2

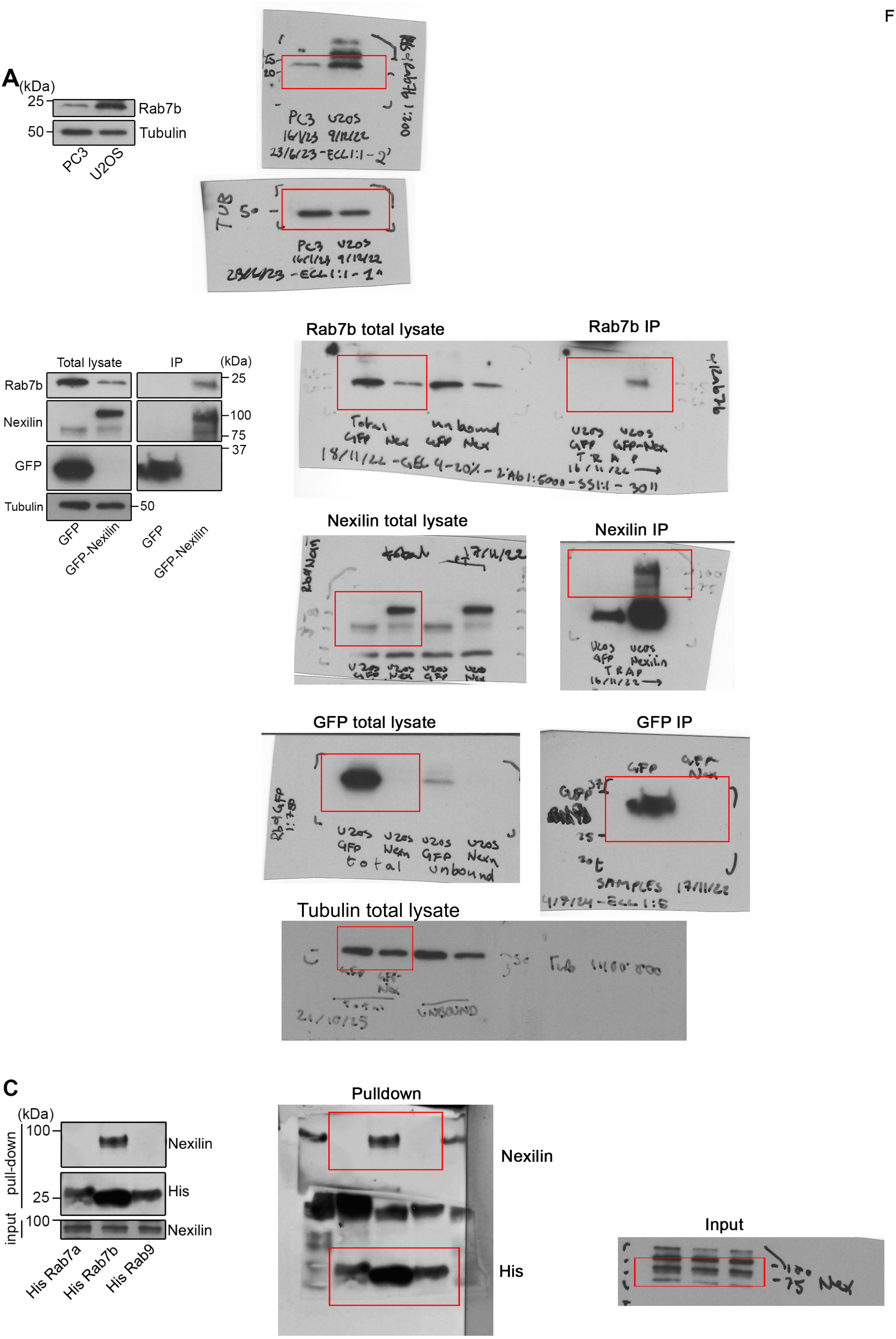
A
(kDa)
25
50
Rab7b
Tubulin
PC3
U2OS
B
Total lysate
IP
(kDa)
Rab7b
Nexilin
GFP
Tubulin
25
100
75
37
50
GFP
GFP-Nexilin
GFP
GFP-Nexilin
Rab7b total lysate
Rab7b IP
Nexilin total lysate
Nexilin IP
GFP total lysate
GFP IP
Tubulin total lysate
C
(kDa)
pull-down
input
100
25
100
Nexilin
His
Nexilin
His Rab7a
His Rab7b
His Rab9
Pulldown
Nexilin
His
Input

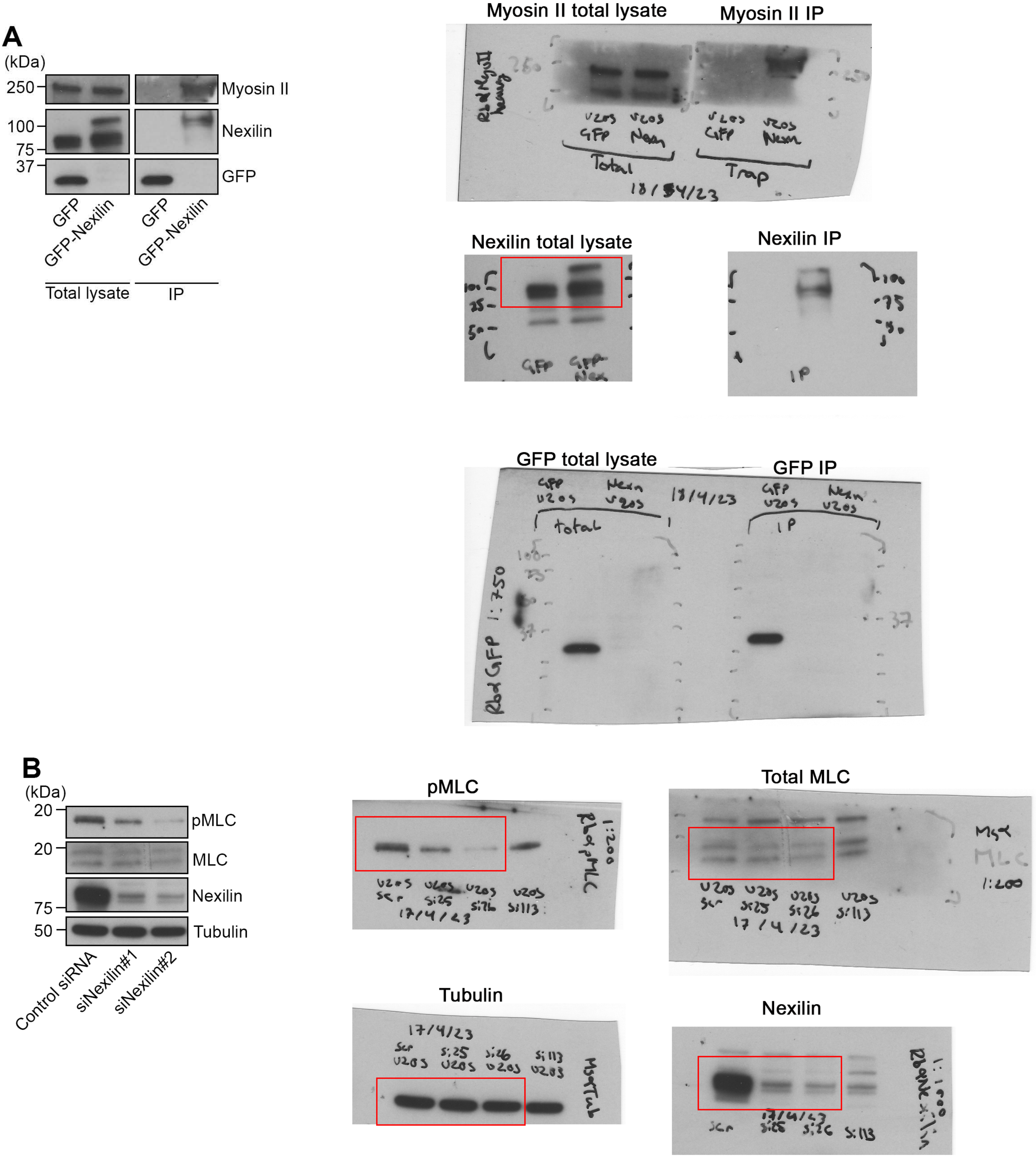
A
(kDa)
250
100
75
37
Myosin II
Nexilin
GFP
GFP
GFP-Nexilin
GFP
GFP-Nexilin
Total lysate
IP
Myosin II total lysate
Myosin II IP
Nexilin total lysate
Nexilin IP
GFP total lysate
GFP IP
B
(kDa)
20
20
75
50
pMLC
MLC
Nexilin
Tubulin
Control siRNA
siNexilin#1
siNexilin#2
pMLC
Total MLC
Tubulin
Nexilin

Figure 3

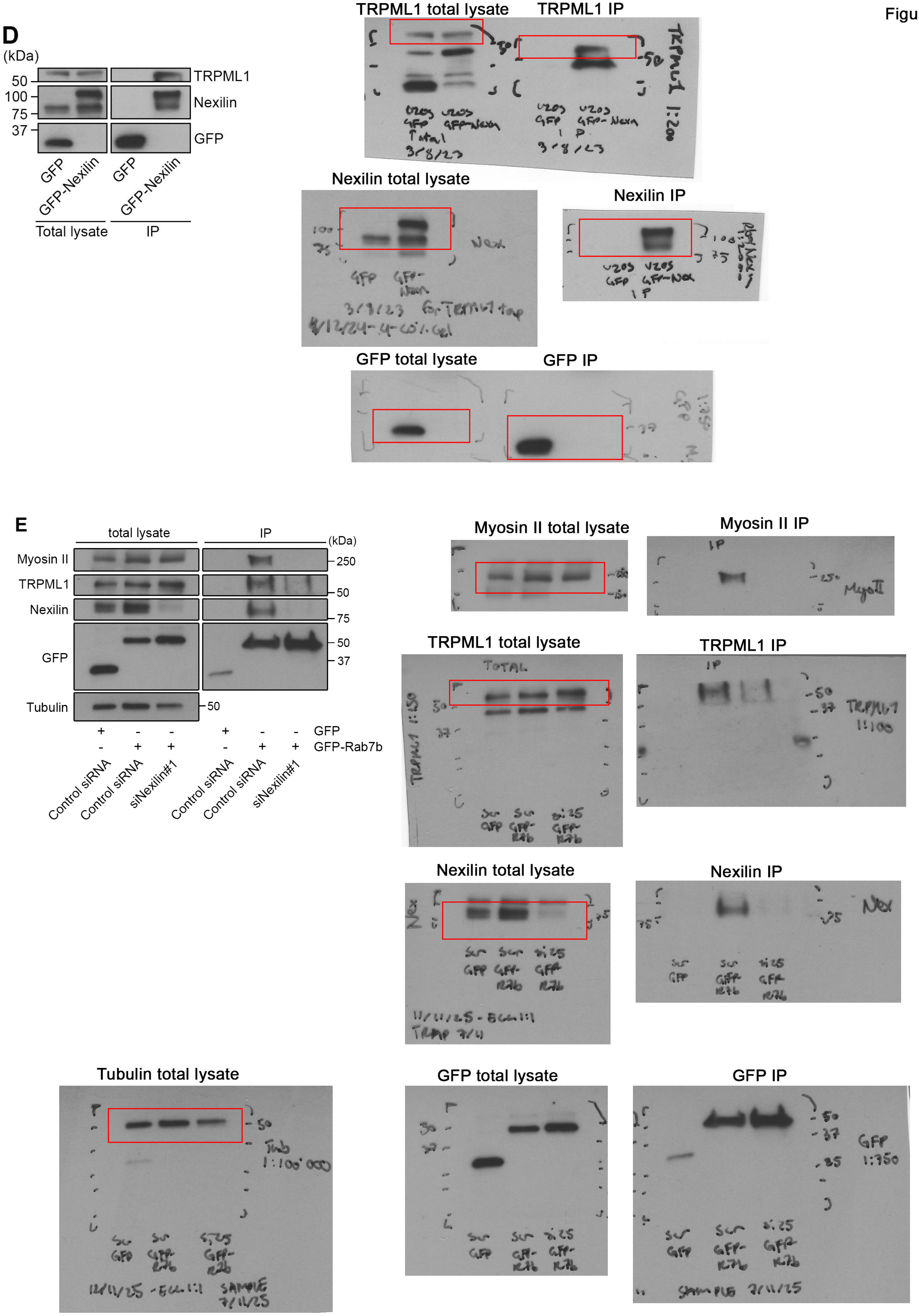
D
(kDa)
50
100
75
37
TRPML1
Nexilin
GFP
GFP
GFP-Nexilin
GFP
GFP-Nexilin
Total lysate
IP
TRPML1 total lysate
TRPML1 IP
Nexilin total lysate
Nexilin IP
GFP total lysate
GFP IP
E
total lysate
IP
(kDa)
Myosin II
TRPML1
Nexilin
GFP
Tubulin
250
50
75
50
37
50
+ - - + - - GFP
- + + - + + GFP-Rab7b
Control siRNA
Control siRNA
siNexilin#1
Control siRNA
Control siRNA
siNexilin#1
Myosin II total lysate
Myosin II IP
TRPML1 total lysate
TRPML1 IP
Nexilin total lysate
Nexilin IP
Tubulin total lysate
GFP total lysate
GFP IP

**B**

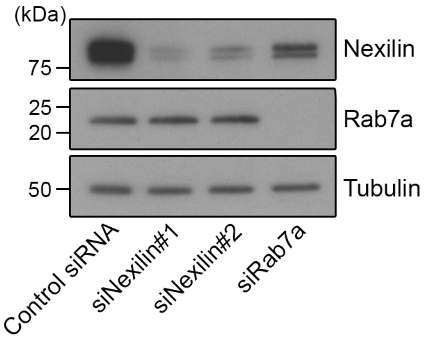


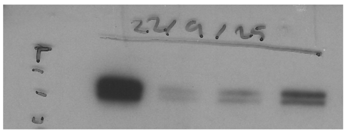


**Nexilin**

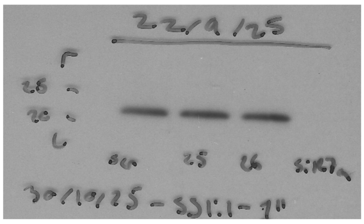


**Rab7a**

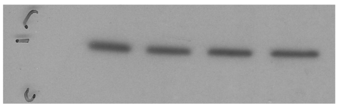

**Tubulin**

Supplementary figure 2

**A**

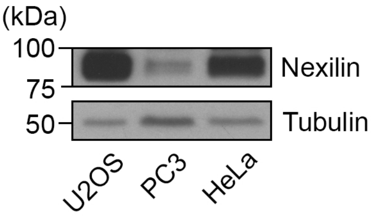


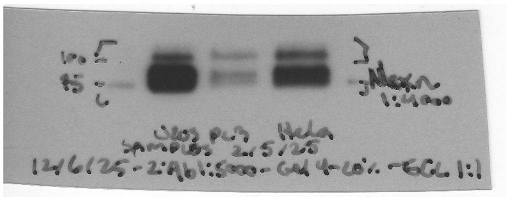


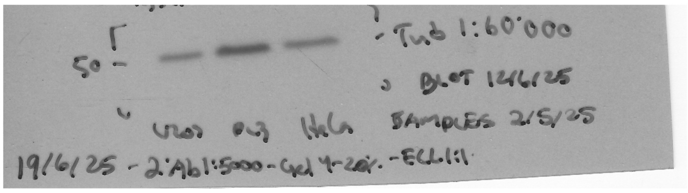


**E**

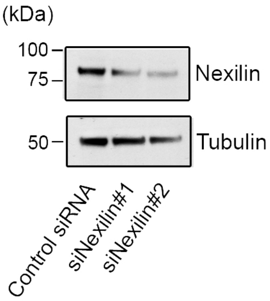


Nexilin

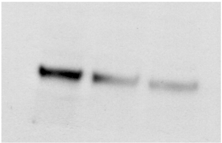

Tubulin

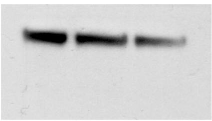

**A**

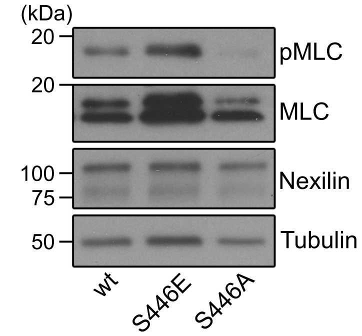


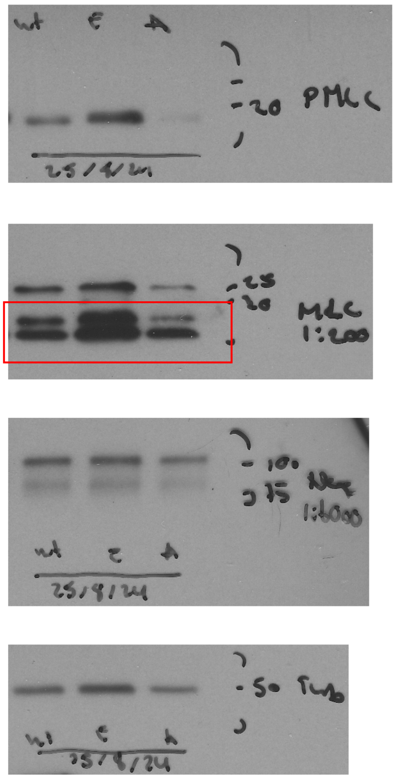

Supplement: Supplementary file 9 — Supplementary Material 9 [file 12964_2025_2628_MOESM9_ESM.pdf]
